# Sex Bias and Maternal Contribution to Gene Expression Divergence in *Drosophila* Blastoderm Embryos

**DOI:** 10.1371/journal.pgen.1005592

**Published:** 2015-10-20

**Authors:** Mathilde Paris, Jacqueline E. Villalta, Michael B. Eisen, Susan E. Lott

**Affiliations:** 1 Department of Molecular and Cell Biology, University of California, Berkeley, Berkeley, California, United States of America; 2 Howard Hughes Medical Institute, University of California, Berkeley, Berkeley, California, United States of America; 3 Department of Evolution and Ecology, University of California, Davis, Davis, California, United States of America; Cornell University, UNITED STATES

## Abstract

Early embryogenesis is a unique developmental stage where genetic control of development is handed off from mother to zygote. Yet the contribution of this transition to the evolution of gene expression is poorly understood. Here we study two aspects of gene expression specific to early embryogenesis in *Drosophila*: sex-biased gene expression prior to the onset of canonical X chromosomal dosage compensation, and the contribution of maternally supplied mRNAs. We sequenced mRNAs from individual unfertilized eggs and precisely staged and sexed blastoderm embryos, and compared levels between *D*. *melanogaster*, *D*. *yakuba*, *D*. *pseudoobscura* and *D*. *virilis*. First, we find that mRNA content is highly conserved for a given stage and that studies relying on pooled embryos likely systematically overstate the degree of gene expression divergence. Unlike studies done on larvae and adults where most species show a larger proportion of genes with male-biased expression, we find that transcripts in *Drosophila* embryos are largely female-biased in all species, likely due to incomplete dosage compensation prior to the activation of the canonical dosage compensation mechanism. The divergence of sex-biased gene expression across species is observed to be often due to lineage-specific decrease of expression; the most drastic example of which is the overall reduction of male expression from the neo-X chromosome in *D*. *pseudoobscura*, leading to a pervasive female-bias on this chromosome. We see no evidence for a faster evolution of expression on the X chromosome in embryos (no “faster-X” effect), unlike in adults, and contrary to a previous study on pooled non-sexed embryos. Finally, we find that most genes are conserved in regard to their maternal or zygotic origin of transcription, and present evidence that differences in maternal contribution to the blastoderm transcript pool may be due to species-specific divergence of transcript degradation rates.

## Author Summary

The early embryonic stage of development is unique as this stage begins under the genetic control of the mother and transitions to genetic control of the zygote. In order to investigate how these developmental specificities have shaped gene expression evolution, we studied the contribution of maternal gene expression to the pool of transcripts in the early embryo and how gene expression differs in early female and male embryos. We find extensive female-bias in gene expression, something not observed in adults. This is largely due to the delay in the establishment of a mechanism that balances gene expression from the differing number of X chromosomes in females and males. The maternal contribution to the transcript pool in the embryo is largely conserved over species, with some exceptions. However, there may also be some species-specific differences in the degradation rate of maternal transcripts, an often-overlooked contributor to variation in transcript level.

## Introduction

To investigate the role of gene expression in development and its evolution, gene expression must be examined at multiple developmental stages across populations or species. The early embryonic stages of development are of special interest because they are genetically controlled by both the mother and the zygote. Indeed in all animals, the earliest embryo is transcriptionally inactive and contains only maternally deposited mRNAs that are progressively eliminated while zygotic transcription takes over. In this study, we focus on two aspects of this handover of developmental control from the mother to the zygote: the activation of zygotic transcription and its regulation and the contribution of maternal mRNA content to embryonic gene expression. First in *Drosophila*, the early embryonic stage is characterized by higher transcript levels in females for many zygotic genes on the X chromosome, indicating incomplete dosage compensation before the canonical MSL-mediated X chromosomal dosage compensation mechanism of transcription is established [1,2]. Second, during the early phases of development, the proportion of maternal mRNAs in the embryo drops rapidly, due to a tightly co-regulated process whereby maternal RNAs are degraded as zygotic RNAs begin to be transcribed (referred to as the maternal to zygotic transition, reviewed in [3]). How these two factors, incomplete X chromosomal dosage compensation and maternal transcripts, contribute to the evolution of embryonic gene expression remains unclear.

Patterns of sex-specific gene expression variation and evolution have been the subject of considerable attention (see [4] for review). In *Drosophila*, there have been a number of genome-wide studies examining gene expression in female and male flies across species, in sex-specific and non sex-specific tissues [5,6], whole adult flies[7,8], and gonadectomized flies (primarily in *D*. *melanogaster* only [9,10]). These studies (and numerous others in other systems) have come to a number of common conclusions, for example, that there are generally more male-biased than female-biased genes [8], and that male-biased genes are fast evolving [5,7,8], as are genes expressed in sex-limited reproductive tissues [11].

Gene expression in embryos has largely not been a part of this discussion of evolution of sex-biased gene expression, as sex-specific transcriptomes of embryos have been previously out of reach. As embryonic development is critical to the fitness of the organism, and the selective pressures and developmental constraints of this stage differ, we hypothesized that some of the patterns observed in adults might differ in embryos. A factor that is of critical importance is the incompleteness of X chromosomal dosage compensation in the early embryo [1,2,12–14]. Additionally, many studies addressing sex-biased gene expression focus on sex-specific tissues as compared to somatic tissues. Many genes expressed in sex-specific tissues (particularly male-specific tissues) are also tissue-specific in expression. The blastoderm embryo has not yet formed tissue layers, and will not form sex-specific tissues until much later in development. Thus genes expressed in this stage, and gene expression changes, are more likely to be pleiotropic than those in sex-specific tissues later in development. However, there are also many genes at the blastoderm stage that are specific to this time of development, so they may be less pleiotropic over developmental time.

Regulation of gene expression in the *Drosophila* blastoderm embryo has long been the subject of fruitful and extensive study. During this stage, widespread activation of zygotic transcription and degradation of maternal transcripts takes place. A number of additional developmental processes also occur during this stage, including cellularization of the previously syncytial embryo, and important steps in both the segmentation of the embryo along the anterior-posterior axis and future development of tissue layers along the dorsal-ventral axis. Despite the considerable resources that have been employed to understand development at this stage, it remains difficult to study in a comparative context. Different species vary in the timing and the duration of developmental stages [15,16], and the tendency of particular species to lay unfertilized eggs [17] or withhold egg deposition until the egg is of an advanced stage [16] contribute to the difficulties of comparative study.

We previously developed a technique to sequence transcriptomes of single *Drosophila* embryos [1], which has facilitated comparative studies of transcript levels across species [2]. With the ability to examine and image single individuals, we are able to obtain samples of precisely the same developmental stage according to embryo morphology. This also avoids concerns of female egg withholding, and we are able to visually identify, and remove, unfertilized eggs. Single embryo methods have also made it possible to examine transcriptome-level data in individual female and male embryos [1,2], rather than the mixed-sex pools that are usually studied.

**Fig 1:**
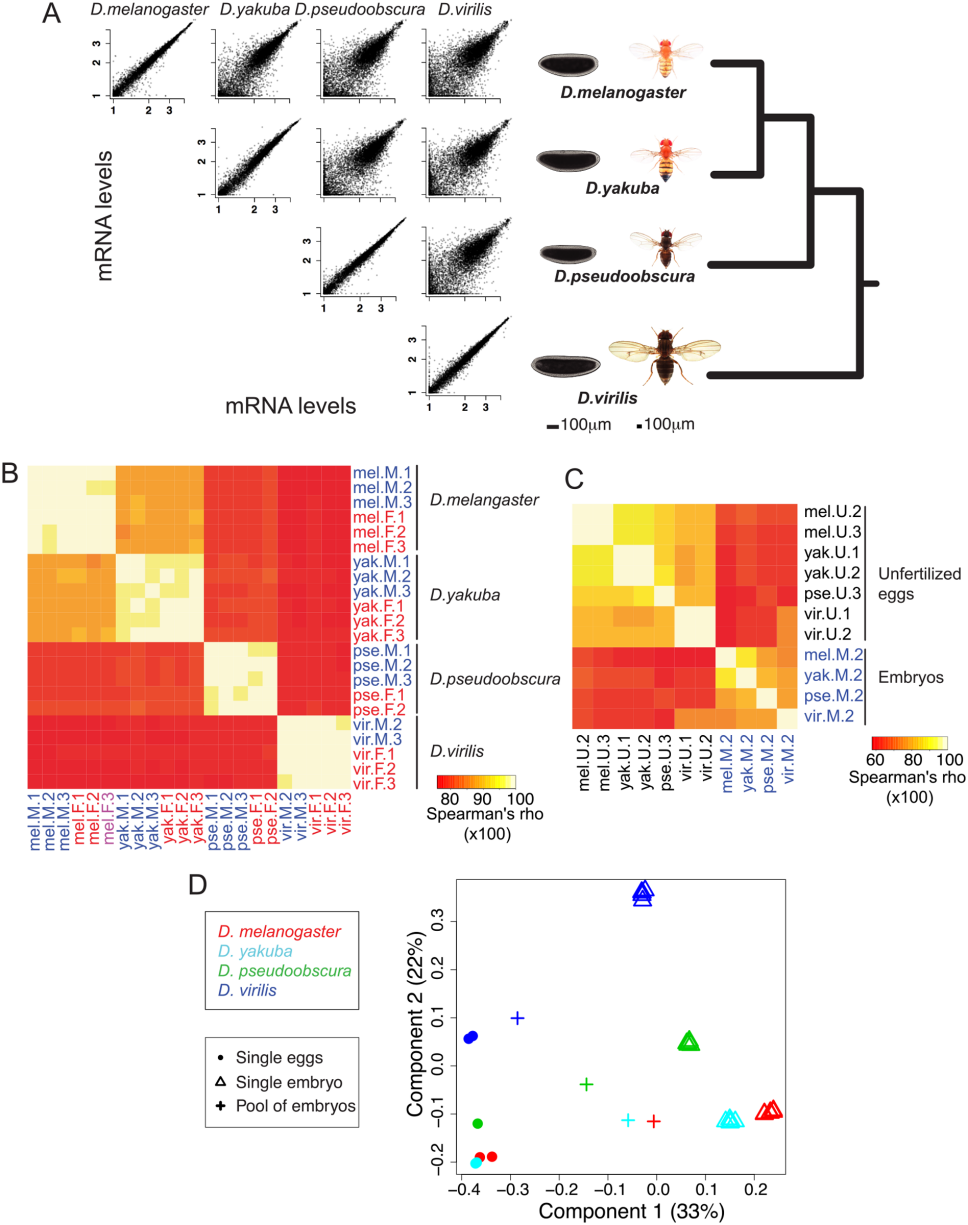
General changes of embryonic gene expression between four *Drosophila* species. **A.** In this paper we compare the genome-wide mRNA levels in blastoderm embryos of four *Drosophila* species *D*. *melanogaster*, *D*. *yakuba*, *D*. *pseudoobscura* and *D*. *virilis*. Pairwise comparisons of mRNA levels between one male replicate in different species show that mRNA levels are overall highly conserved. As a comparison, scatter plots of two male replicates are shown for each species. **B.** Spearman correlations between mRNA levels of the 22 tightly staged embryos of our dataset (2–3 males/2–3 females per species). Correlations decrease with increasing phylogenetic distance. **C**. Spearman correlations between mRNA levels of the seven unfertilized eggs (1–2 per species) and a representative embryo sample from each species (“Male replicate 2”). Correlations are highest within eggs and embryos than between eggs and embryos. For **B** and **C**, the Spearman correlations are shown in percentages (so ranging from 0 to 100). **D.** Correspondence analysis (COA) of expression levels in the single eggs and embryos. Values for pools of embryos were projected to the COA. The proportion of the variance explained by the first components of the correspondence analysis is indicated in parentheses. Samples separate along first axis mainly according to developmental stage while they separate along second axis mainly according to species.

In order to determine the variation and evolutionary patterns of sex-specific gene expression across *Drosophila* species, we chose four species spanning the evolutionary history of the genus, *Drosophila melanogaster*, *Drosophila yakuba*, *Drosophila pseudoobscura*, and *Drosophila virilis* (Fig 1A). These species diverged from 5 to 40 million years ago [18], yet to a large degree, are conserved in their early embryonic gene expression [19].

## Results

To characterize the sex-specific transcriptomes of individual blastoderm *D*. *melanogaster*, *D*. *yakuba*, *D*. *pseudoobscura*, and *D*. *virilis* embryos, we collected single embryos, imaged them to determine developmental stage, and extracted RNA and DNA for subsequent analysis (as in [1,2]). We chose the end of blastoderm stage (stage 5, mitotic cycle 14), when cellularization has been completed, but prior to gastrulation, as a precise timepoint observable from morphology, so that it would be a homologous timepoint across species. We used the extracted DNA in a PCR assay to determine the sex of the each embryo, using multiple Y-chromosome specific primers designed independently for each species (see Methods). We used RNA from individual female embryos (three replicates) and individual male embryos (three replicates) from each species (for a total of six libraries per species) to make libraries for RNA sequencing, without amplification of input RNA as previously described [1]. After sequencing, one female *D*. *pseudoobscura* embryo and one *D*. *virilis* male embryo were removed from analysis due to low data quality. In addition, we also harvested individual newly deposited unfertilized eggs (referred to hereafter simply as “eggs”, as opposed to the blastoderm stage individuals, referred to as “embryos”) from each species, and used them to construct single egg mRNA-Seq libraries (two libraries for *D*. *melanogaster*, *D*. *yakuba* and *D*. *virilis* and one library for *D*. *pseudoobscura*). Libraries were sequenced on an Illumina HiSeq 2000 DNA Sequencer.

We aligned reads to the *D*. *melanogaster* reference annotation (Flybase r5.39) or to *D*. *yakuba*, *D*. *pseudoobscura* or *D*. *virilis* annotations produced in a previous study [19] using Bowtie2 [20]. Transcript levels were inferred using eXpress [21] and gene levels were computed as the sum of corresponding transcript levels. Gene expression was normalized so that the 75^th^ percentile levels of genes located on autosomes (Müller elements B, C, and E) was identical between samples, then log transformed (log_10_(normalized FPKM+1)). Additional normalization procedures were also performed (median, 95^th^ percentile, TMM, quantile normalizations after eXpress as well as no further normalization other than the one performed by eXpress) and they gave essentially the same results (see the Methods section for further details). Below we focus on 5,985 genes that were expressed in at least one condition (egg, male or female embryo) in any species (normalized FPKM averaged over all replicates of a condition above 1 before log transformation) and for which we could establish a clear 1:1:1:1 orthology [19].

Pairwise plots of transcript levels show highly reproducible sampling within a species (plots along the diagonal in Fig 1A), with Spearman correlation coefficients of 0.98–0.99 (Fig 1B). There is also a high level of conservation of transcript levels in the blastoderm embryo across species. These correlations of transcript levels decrease with increasing evolutionary distance, for example, when comparing *D*. *melanogaster* to the other species, the highest correlation is with *D*. *yakuba* (median correlation of ~0.89), then with *D*. *pseudoobscura* (~0.81), and *D*. *virilis* (~0.78) (S1 Fig). Correlations for unfertilized eggs between these species are systematically slightly higher than that for embryos: again when comparing *D*. *melanogaster* eggs to the other species, median correlations are ~0.92 for *D*. *yakuba*, ~0.85 for *D*. *pseudoobscura* and ~0.83 for *D*. *virilis* (Fig 1C and S1 Fig). These higher correlations may imply more stringent developmental constraints on maternal transcript deposition (eggs), than early zygotic transcription (blastoderm embryos).

Our single embryo approach differs from most studies that have compared transcript levels genome-wide in pools of *Drosophila* embryos, typically collected over a two-hour window [22,23]. Pooling has theoretical disadvantages over a single embryo approach, because pooling combines mRNA content from embryos of various ages, which may bias subsequent analyses and obscure any meaningful comparative results. We had previously created an RNA-seq dataset obtained from pools of late-stage 4 to mid-stage 5 embryos in *D*. *melanogaster*, *D*. *yakuba*, *D*. *pseudoobscura and D*. *virilis* [19]. Having collected both the pooled [19] and individual egg/embryo (this study) datasets in the same four species, we compared them to more accurately determine the nature of the potential systematic “pooling” bias. We first compared gene levels between our single eggs and embryos through a correspondence analysis (COA, Fig 1D). As shown in Fig 1D, the correspondence analysis separates the samples by stage (first axis) and by species (second axis). We then projected on the COA expression data from pools of embryos spanning the end of stage 4 to mid-stage 5 that had been processed the same way as the single embryo/egg samples.

The pooled samples fall on the same position as single embryos and eggs along the second (species) axis but are intermediate between eggs and embryos on the developmental stage axis. In particular, the *D*. *virilis* pool sample resembles single egg samples more than other species (accordingly, levels from this sample are highly correlated with levels from eggs, Fig 1C), which is likely due to both higher proportion of undeveloped eggs in this species and differences in the distribution of stages within the collection window. Indeed, mRNA molecules were harvested from pools of embryos at any stage between late stage 4 and early stage 5, and not at a single time point. The distribution of time points within the collection window is likely to vary between species, and as a consequence the “averaged” levels from the sequenced mRNA pool do not represent the same “average” stages in the different species. In summary, pooling embryos of several developmental stages creates a confounding factor between developmental stage and species. Consequently, comparative analyses based on such pools of embryos may falsely attribute developmental differences as species differences and should be avoided as much as possible. In addition and unless specified, we averaged transcript levels over female embryo replicates, male embryo replicates, and egg replicates.

### General patterns of sex-bias across species

We define a sex-biased gene as a gene with a higher transcript level in females compared with males or *vice versa*, as is used with studies of later stages of development when canonical dosage compensation is fully established on the X chromosome. As discussed in greater detail below, much of what we define as female-bias is likely due to lack of complete dosage compensation of the X chromosome (Muller A) at this stage of development. Our previous study showed that roughly half of zygotic genes on the X chromosome in *D*. *melanogaster* have equal transcript levels in females and males at this developmental stage, prior to the onset of MSL-mediated dosage compensation, and half are higher in females, suggesting no or partial dosage compensation [1]. However, with this study, we lack the allele-specific data necessary to validate whether transcripts are maternal or zygotic in origin, which is necessary for quantifying dosage compensation (maternal transcripts originate only from females, so no dosage imbalance exists for these templates). Thus, it is difficult to make assumptions about the expected compensation status or expression relative to dosage for genes at this point in development. We use the definition of sex-bias as having a higher transcript level in one sex, but make no assumption as to the mechanistic basis of this bias. This also permits comparison to the large body of work examining sex-specific transcript levels in later developmental stages.

**Fig 2:**
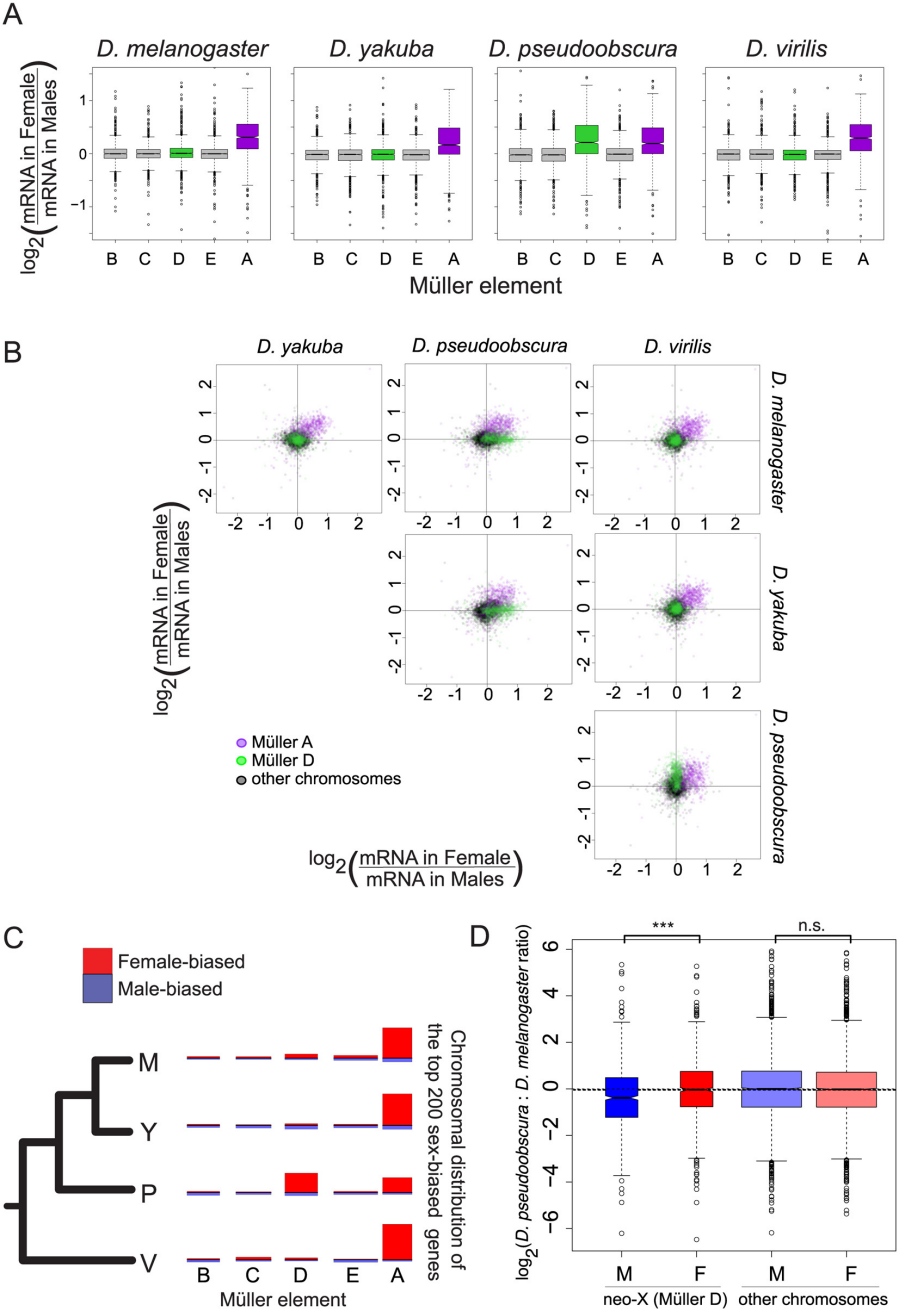
Gene expression on the neo-X (Müller D element) in *D*. *pseudoobscura* does not show complete dosage compensation. **A**. Gene expression on sex chromosomes shows partial dosage compensation in all species. The sex ratio (female/male) was calculated for each gene and compared between chromosomes. Genes from all autosomes show similar expression in both sexes (log_2_ ratio close to 0) while genes located on the Müller A element (purple) in all species and on the Müller D element (green) in *D*. *pseudoobscura* show a systematic bias towards higher ratio. **B.** Plot of pairwise sex ratio, the ratio of female transcripts to male transcripts, between species. The Müller A (purple) element is female-biased in all species, whereas the Müller D (green) is female-biased only in *D*. *pseudoobscura*. **C.** The top 200 most sex-biased genes are mostly female-biased, and are mostly located on sex chromosomes: the Müller A element in all species, and Müller D in *D*. *pseudoobscura*. Each chromosome was drawn with a length proportional to gene content. The height of red bars drawn above each chromosome and blue bars drawn below each chromosome represents the proportion of genes from this chromosome that are female-biased and male-biased, respectively. **D.** The decrease in sex ratio for genes located on the Müller D element in *D*. *pseudoobscura* is due to a decrease in male expression in *D*. *pseudoobscura* rather than an increase in females. We compared mRNA levels between *D*. *pseudoobscura* and *D*. *melanogaster* males and females for genes located either on the Müller D element or on other elements. Genes located on the Müller D element had similar mRNA levels in females but exhibited lower mRNA levels in *D*. *pseudoobscura* males compared to *D*. *melanogaster* males. ***: Wilcoxon test p-value < 0.001; n.s.: Wilcoxon test p-value > 0.05.

Genome-wide, transcript levels are largely the same between female and male blastoderm embryos (Fig 1B). But sex chromosomes, and the incomplete nature of X chromosomal dosage compensation at this stage (prior to the onset of MSL-mediated dosage compensation, [1,2,12,13]), contribute to sex-specific expression differences. Generally, autosomal genes have similar, non-sex biased, transcript levels across species (Fig 2A–2C). In all species, at this period of developmental time, Müller A (the shared X chromosome) appears to have incomplete dosage compensation (Fig 2A). Genes from this shared sex chromosome are largely similarly female-biased across species (purple dots in Fig 2B). There is no sex-bias in maternal deposition of mRNAs, as these RNAs are deposited before the eggs are fertilized by X or Y bearing sperm. As around half of the transcripts at blastoderm stage are maternal in origin, the female-bias in genes at this time period presumably originates with zygotically expressed genes. The other striking pattern of sex-bias is on the neo-X chromosome in *D*. *pseudoobscura*, where Müller D has undergone chromosomal fusion with Müller A [24]. Müller D in *D*. *pseudoobscura* shows patterns of sex-bias similar to Müller A, with female-bias in transcript levels, indicating incomplete dosage compensation (Fig 2A–2C). This increase in female-bias is primarily due to a decrease in male expression in the *D*. *pseudoobscura* lineage (Fig 2D, Wilcoxon test p-value < 1.2 10^−6^ for all normalizations), which is consistent with females having two copies of Müller D as in the rest of the species where this element is an autosome, whereas *D*. *pseudoobscura* males only have a single copy.

Overall, there are more female-biased genes than male-biased genes, either proportionally of all genes with sex-bias (Fig 2C), or in total (S1 Table) at various cutoffs, for all species. This is in contrast to studies in adults, where only *D*. *pseudoobscura* was thought to have an overall higher level of female-biased genes than male-biased genes [8]. Presumably, our result differs due to incomplete dosage compensation at this stage, which leads to a greater proportion of female-biased genes. Similar to studies in adults [8], we observe that the magnitude of sex-bias is generally greater for male-biased genes (see S1 Table for various sex-bias cutoffs and Wilcoxon test p-values). While we fail to detect a general relationship between sex-bias and expression level, we do find that the most highly expressed genes on the X chromosomes (Müller A in all species, plus Müller D in *D*. *pseudoobscura*) are more likely to have the same transcript abundance in females and males (S2 Fig, species-specific Wilcoxon test p-values all < 2.5x10^-9^, comparing X chromosomal genes with a log10 transcript level of <2 to those >2). Most genes with high transcript levels at this stage are largely maternal in origin in *D*. *melanogaster*, which can potentially explain this result (the trend is likely to be similar in other species).

### Species-specific divergence of sex-bias

**Fig 3:**
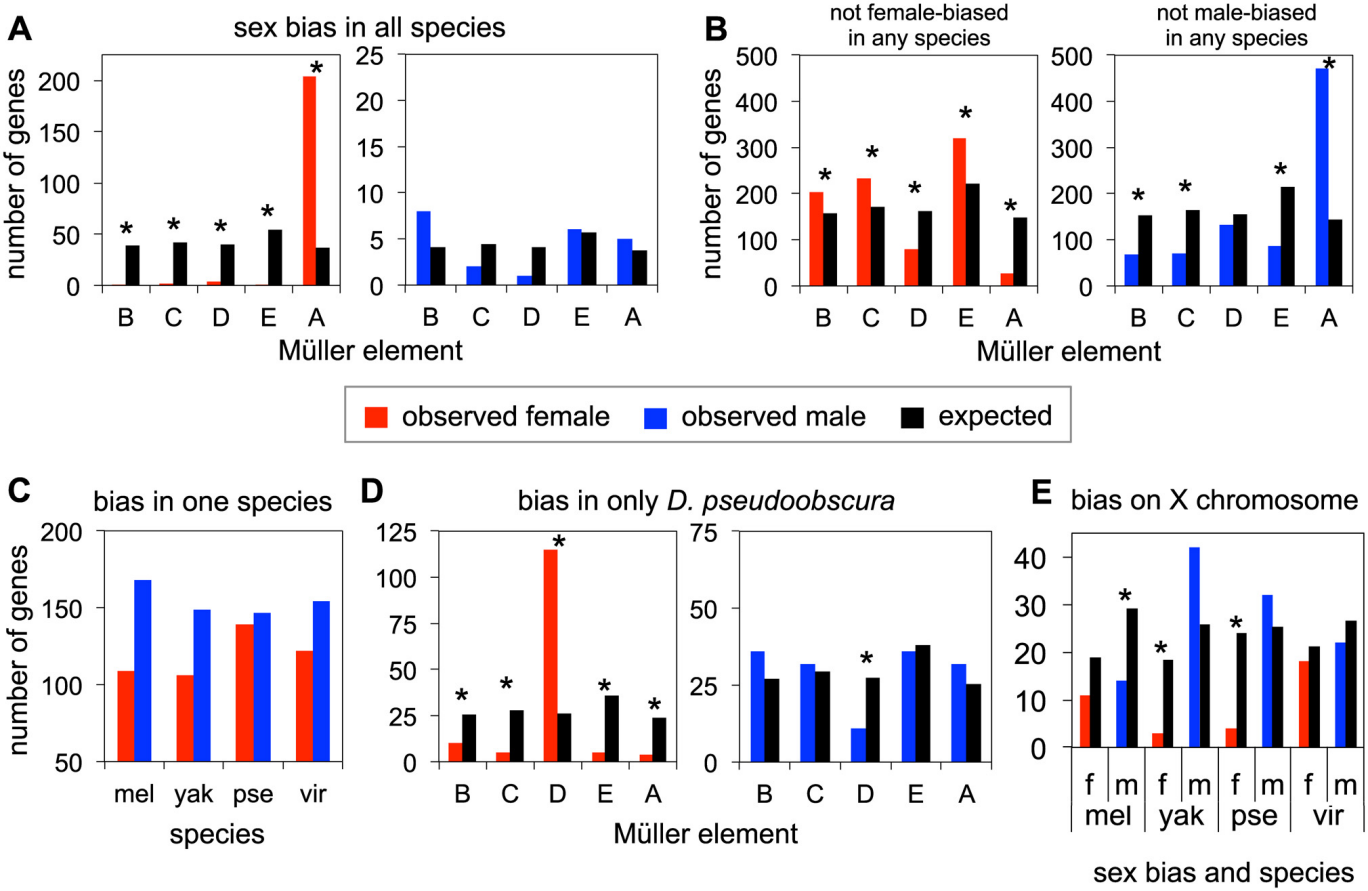
Patterns of sex-bias over genes. **A.** Numbers of genes that are female-biased (red) or male-biased (blue) in all four species across all chromosomes, as compared to the expected chromosomal distribution (black) proportional to number of genes on each chromosome. Note the difference in scale on the Y-axis between the female-bias and male-bias comparisons. **B.** Numbers of genes that are not female or male-biased in any of the four species, on each chromosome. **C.** Genes that are female or male-biased in only one species (and unbiased in the other three). **D.** Observed and expected chromosomal distributions of genes that are female or male-biased only in *D*. *pseudoobscura*. **E.** Genes that are female or male-biased on the X chromosome. Statistically significant results (p< 0.05) from chi-squared tests indicated with *, see S2–S5 Tables.

In order to identify species-specific changes in sex-bias, we constructed rankings of sex-bias for all genes, within each species. The rank comparison of transcript levels between females and males of the same species provides a measurement largely unaffected by concerns of annotation or genome quality that can plague the analysis of quantitative mRNA levels between species. These rankings were used to investigate patterns of sex-bias across species, and across chromosomes. Female-bias and male-bias are then defined as being in the top or bottom percentiles of the sex rankings. Fig 3 presents results using the top and bottom 20% of sex-biased genes in each species, and qualitatively similar results for a range of cutoffs are presented in S2–S6 Tables. Genes reported as not sex-biased are in the middle 20% of the distribution (40–60%), and ranges of cutoffs for non-sex biased genes are also presented in S2–S6 Tables.

There are a large number of genes that show female-bias across all four species, compared to the small number that show male-bias across all species (S2 Table). We next examined the chromosomal distribution of biased genes. The genes that show female-bias in all four species are highly enriched on Müller A, relative to an expected uniform chromosomal distribution, and this excess is highly significant (chi squared test, p = 2.06x10^-31^, Fig 3A and S2 Table). In contrast, the number of genes showing male-bias across all four species is quite small, and there is no significant pattern of chromosomal enrichment (Fig 3A and S2 Table). Thus embryos, like later developmental stages, have a largely feminized X chromosome, but show no evidence for demasculinization on this chromosome.

Next, we examined genes with no female-bias or no male-bias across all four species. The total number of genes across all four species showing no female-bias is remarkably similar to those showing no male-bias (S3 Table). Non-female biased genes are defined as those whose female to male transcript level ratios rank in the bottom 60% of the distribution, non-male biased genes are those whose female to male transcript level ratios rank in the top 60% (see S3 Table for other cutoffs). The similar number of genes having no female- or no male- bias in all four species is presumably a product of higher numbers of genes that are female-biased in all four species, and the higher number of genes that are male-biased in a single species. The chromosomal distribution of non-sex biased genes is largely the inverse of the patterns of sex-biased genes (Fig 3B). Genes showing no female-bias in any of the four species are underrepresented on Müller A (p = 1.28x10^-22^, S3 Table), and to a lesser extent, Müller D (p = 2.75x10^-8^, S3 Table), compared to the expected distribution. In contrast, genes that have no male-biased expression in any of the four species are highly overrepresented on Müller A (p = 3.09x10^-64^, S3 Table).

Analysis of genes that show a lineage-specific pattern of sex-bias is dominated by the female-bias (and lack of male-bias) of Müller D (XR) in *D*. *pseudoobscura*. To determine lineage specific genes, we used the sex-bias rankings in each species to determine those that were female or male-biased in one species, and unbiased in the other three species (see Methods, and S4 and S5 Tables for additional sex-bias and not sex-biased cutoffs). We find overall a higher number of genes with male-bias in only one species compared to genes that are female-biased in only one species. *D*. *pseudoobscura* has the highest level of lineage specific female-bias, and is the only species where the number of female-biased genes approaches male-biased genes (Fig 3C and S4 Table). Examining the observed chromosomal distribution of genes showing sex-bias only in *D*. *pseudoobscura* (Fig 3D and S5 Table), we observe a significant enrichment of female-biased genes on Müller D (chi squared test p = 9.26 x 10^−15^), compared to the expected distribution if sex-biased genes were randomly distributed across chromosomes. Conversely, we find a deficit of male-biased genes on Müller D (chi squared test, p = 0.01). This particular distribution of *D*. *pseudoobscura*-specific sex-biased genes is likely due to the chromosomal rearrangement that converted Müller D into a sex chromosome, broadly caused a decrease of gene expression in males for this chromosome, and thus an increase of female-biased genes and a decrease of male-biased genes.

Chromosomal distributions for lineage-specific sex-biased genes in the remaining species show no significant patterns, with the possible exception of Müller A (Fig 3E and S5 Table). However no systematic excess of species-specific sex-biased genes was detected on Müller A, whereas Müller A contains an excess of ancestrally female-biased genes. We observe both excesses and deficiencies of male-biased genes on Müller A, as well as deficiencies in female-biased genes, though only in some lineages. Though underrepresentation of female-biased genes and overrepresentation of male-biased genes in *D*. *pseudoobscura* on Müller A are driven by Müller D (i.e. male-biased genes are overrepresented on Müller A due to the avoidance of male-bias on Müller D), patterns in other species are potentially of interest. *D*. *melanogaster* has a deficiency of both female and male-biased genes on Müller A (Fig 3D), though only the male-biased genes met the threshold for statistical significance at almost all cutoffs (S5 Table). *D*. *yakuba* also has a deficiency of female-biased genes on Müller A (Fig 3D, chi sq. p = 0.002). Finally we found that species-specific female-bias (genes that have female-biased transcript levels in one species, but not the other three) is usually due to lower transcript abundance in males rather than higher transcript levels in females (S6 Table). Similarly, lineage-specific male-bias is primarily due to lower female transcript levels, though in some comparisons, male-bias is also observed to be due to an increase in male transcript level, with or without a change in female transcript level (see S6 Table for more detail).

### Chromosome-specific divergence of mRNA levels

**Fig 4:**
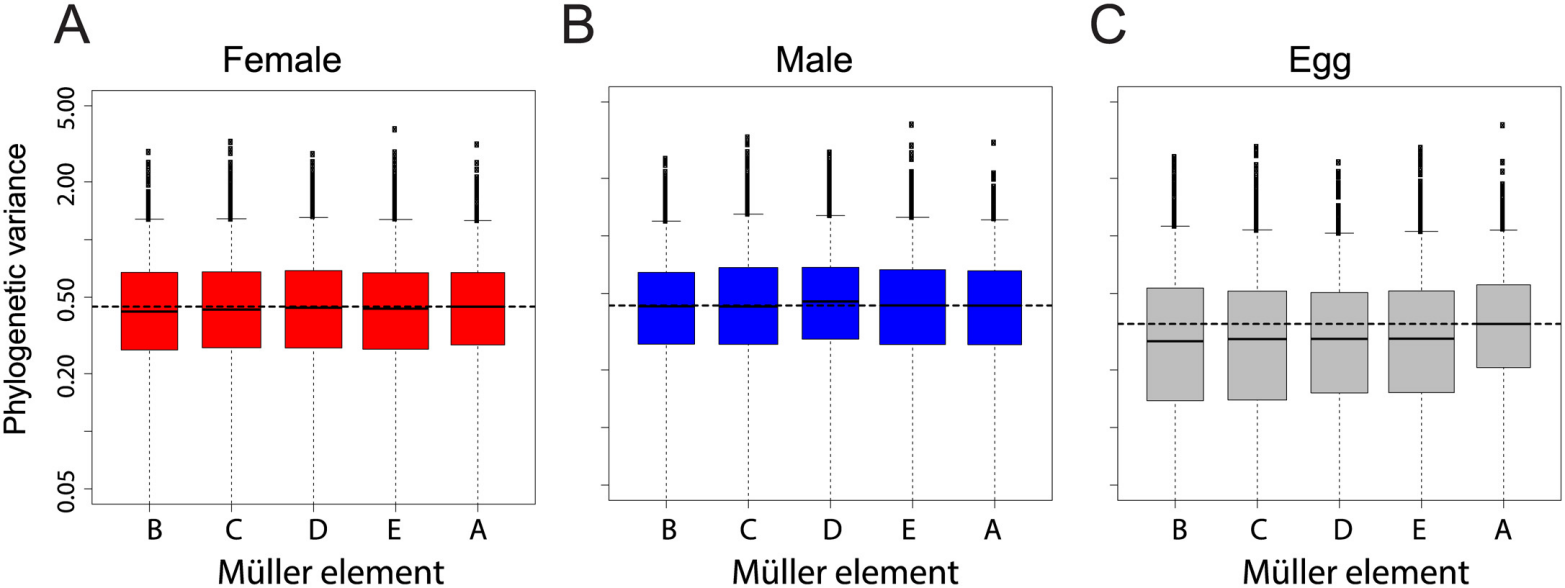
Chromosomal distribution of inter-species gene expression divergence in females (A), males (B) and eggs (C). Divergence was measured as the variance of transcript levels along the *Drosophila* tree according a Brownian motion model. Horizontal dashed line represents the median variance on chromosome X. Chromosome had no effect on variance in females (ANOVA p-value > 0.48), it had a small and non-significant effect in males (ANOVA p-value>0.05), likely due to the higher variance for genes located on the neo-X. Chromosome had a significant effect on variance in eggs, likely due to the higher variance for genes located on the X (ANOVA p-value<0.05).

Gene expression linked to the X chromosome has been shown to evolve faster than for other chromosomes in *Drosophila* adults [25], and recently in embryos [26]. We examined our dataset for the same “faster-X” signal. We measured gene-wise expression divergence as the variance of mRNA levels for each gene along the *Drosophila* tree according a Brownian motion model (see Methods). We found no chromosome effect on phylogenetic variance of transcript abundance in females (Fig 4A, ANOVA p-value > 0.48) and a small and not significant chromosome effect in males (ANOVA p-value > 0.05), likely due to expression divergence of genes located on Müller element D in the *D*. *pseudoobscura* lineage (neo-X, Fig 4B). Chromosome effect was strongest for unfertilized eggs, in which genes located on Müller element A (X chromosome) showed a higher phylogenetic variance than genes located on other chromosomes (Fig 4C, ANOVA p-value < 0.05).

We confirmed this result using an alternative method that considers the divergence of gene expression using Spearman's rank correlation coefficients between species across chromosomes (as in [25,26], a lower correlation is interpreted as higher divergence). In males and females, correlation was mostly similar between chromosomes and Müller A in particular did not show a lower trend (S3 Fig, blue and red boxes). Bootstrap support for a more divergent gene expression of Müller A compared to other chromosomes was low when comparing *D*. *melanogaster*, *D*. *yakuba* and *D*. *virilis* (~7 to 35%). It was higher for comparisons including *D*. *pseudoobscura* (~60–80%). Only in eggs did bootstrap support for divergence on the Müller A reach 95% (between ~45% and 99%, S3 Fig, grey). For all pairwise comparisons, bootstrap support for a more divergent gene expression of Müller A was systematically much higher in eggs than in embryos (with the extreme case of the *D*. *melanogaster-D*. *yakuba* comparison with a bootstrap support of 86% in eggs and ~8% in embryos, S3 Fig). Overall both methods gave similar results: gene expression on the X chromosome is faster than on other chromosomes for eggs only. We note that previous studies were based on expression levels from pooled samples of embryos collected at various time points during development. We reanalyzed this dataset and found that the faster-X effect was most pronounced for the earliest time points (S4 Fig and S5A Fig), that contain the highest proportion of maternally deposited transcripts (S5B Fig). It is thus likely that some of the reported embryonic faster-X evolution in embryos is occurring in maternally deposited transcripts (and thus in adult females) rather than in embryos. Other systematic bias that may account for the discrepancy between our results and others were tested and are described in the supplementary material. Overall, our dataset does not support the hypothesis of a faster-X effect on gene expression in *Drosophila* embryos but supports a faster-X effect in eggs.

### Maternal contribution to transcript level divergence

All the early processes in embryonic development are controlled by maternally deposited mRNAs and proteins, and later, zygotic transcription is activated while maternal gene products degrade. The stage under examination in this study, the end of blastoderm stage, occurs after the first widespread activation of zygotic transcription. However, a large number of transcripts (~50% in *D*. *melanogaster*) are still maternal in origin. Any divergence we observed in transcript level between species could be due to differences in maternal transcripts or zygotic transcripts, and additionally, differences in zygotic transcripts could be due to changes in upstream maternal factors.

**Fig 5:**
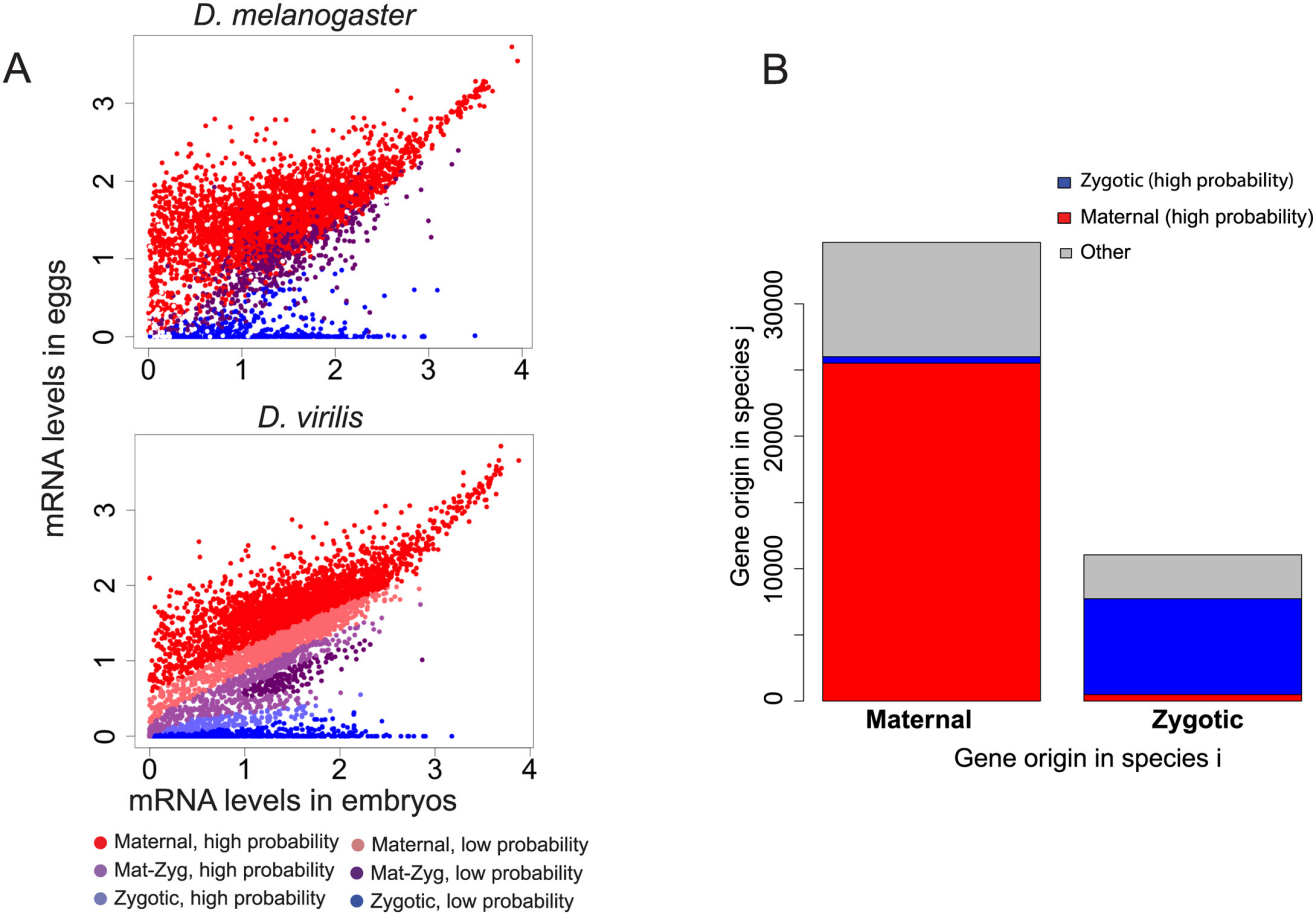
Evolution of the origin of an mRNA transcript in the embryo. mRNAs in the blastoderm embryo can have two possible origins: they were either maternally deposited into the egg and persisted until this stage, or they were actively transcribed in the embryo. This means that genes can be classified in three categories: purely maternal genes (called “Mat”), purely zygotic genes (called “Zyg”), or genes for which some molecules were deposited and some were zygotically transcribed (called “Mat-Zyg”). **A.** This classification has been previously established in *D*. *melanogaster* [1]. mRNA levels tend to partition between the different classifications on a 2D plot comparing eggs and embryos. We learned an SVM on the *D*. *melanogaster* dataset and used it to predict gene classification in the other species. Statistics on the SVM are given in S7 Fig and show that maternal and zygotic genes can be well classified with high confidence, whereas mat-zyg genes are classified with poor success. Fig 5A shows the SVM results for *D*. *virilis*. Similar plots are given for *D*. *yakuba* and *D*. *pseudoobscura* in S8 Fig. Each classification was associated a confidence value, as “high” or “low”. **B.** We compared whether most genes have a conserved origin (“Mat <-> Mat”, “Mat-Zyg <-> Mat-Zyg” or “Zyg <-> Zyg”) or have switched origins between any two species. We only reported changes between maternal and zygotic categories classified by the SVM with high probabilities: “Mat <-> Zyg”. Other categories were merged into “other” (Mat-Zyg classifications and any SVM classifications associated with low probabilities). We found that most genes have a conserved origin, although a non-negligible fraction of genes have different origins (e.g. maternal in one species and zygotic in another).

Because of the importance of maternal transcripts at this stage, we investigated the role of maternal genes in shaping divergence of transcript levels in embryos between species. From a previous experiment in *D*. *melanogaster* embryos including the blastoderm stage, using allele-specific mRNA-sequencing to distinguish maternal transcripts from zygotic transcripts [1], we established a list of genes that could be classified in three categories: genes whose transcripts were only deposited maternally, but not transcribed zygotically, genes with only zygotic transcription and no maternal contribution, and genes whose transcripts are both maternally deposited and zygotically transcribed. These classes are thereafter referred as “Mat”, “Zyg” or “Mat-Zyg”. We observed that maternal and zygotic genes (as predicted by [1]) segregate as expected in eggs and embryos: transcript levels for maternal genes are high in eggs, and transcripts levels for zygotic genes are largely absent in eggs and high in the embryo samples. Accordingly areas containing only “maternal” or “zygotic” genes are clearly visible on a 2D scatterplot comparing gene levels for egg *versus* embryo (Fig 5A). We used these expression values to learn the origin of a transcript among the three possible origins “Mat”, “Zyg” or “Mat-Zyg” using only our gene expression dataset. We randomly divided the 5408 *D*. *melanogaster* genes for which we knew the origin into a training set of 3606 (2/3) genes and a testing set of the remaining 1802 (1/3) genes. We then used the training set to learn a support vector machine (SVM) model from the egg and embryo expression values for each of the three classes of genes. The classification for each gene (“Mat”, “Zyg” or “Mat-Zyg”) was given a probability. The SVM accuracy was assessed based on the number of test genes that had been correctly or incorrectly classified with high probability (above 0.8, 0.8, and 0.7 for “Mat”, “Zyg” and “Mat-Zyg” genes respectively). We found that SVM classification was very accurate for maternal and zygotic genes and performed poorly for “Mat-Zyg” genes (S7 Fig). 96% of the maternal genes were correctly classified (1166 / 1211) and only eight maternal genes were classified as zygotic, all with low probability (none with high probability). 96% of the zygotic genes were correctly classified (308 / 320) and none were classified as maternal. Only 35% of the “Mat-Zyg” genes (93 / 268) were correctly classified. Consequently we mostly focused on predictions of maternal and zygotic genes for subsequent experiments and applied the SVM on our gene expression dataset to predict transcript origin for the other species.

**Fig 6:**
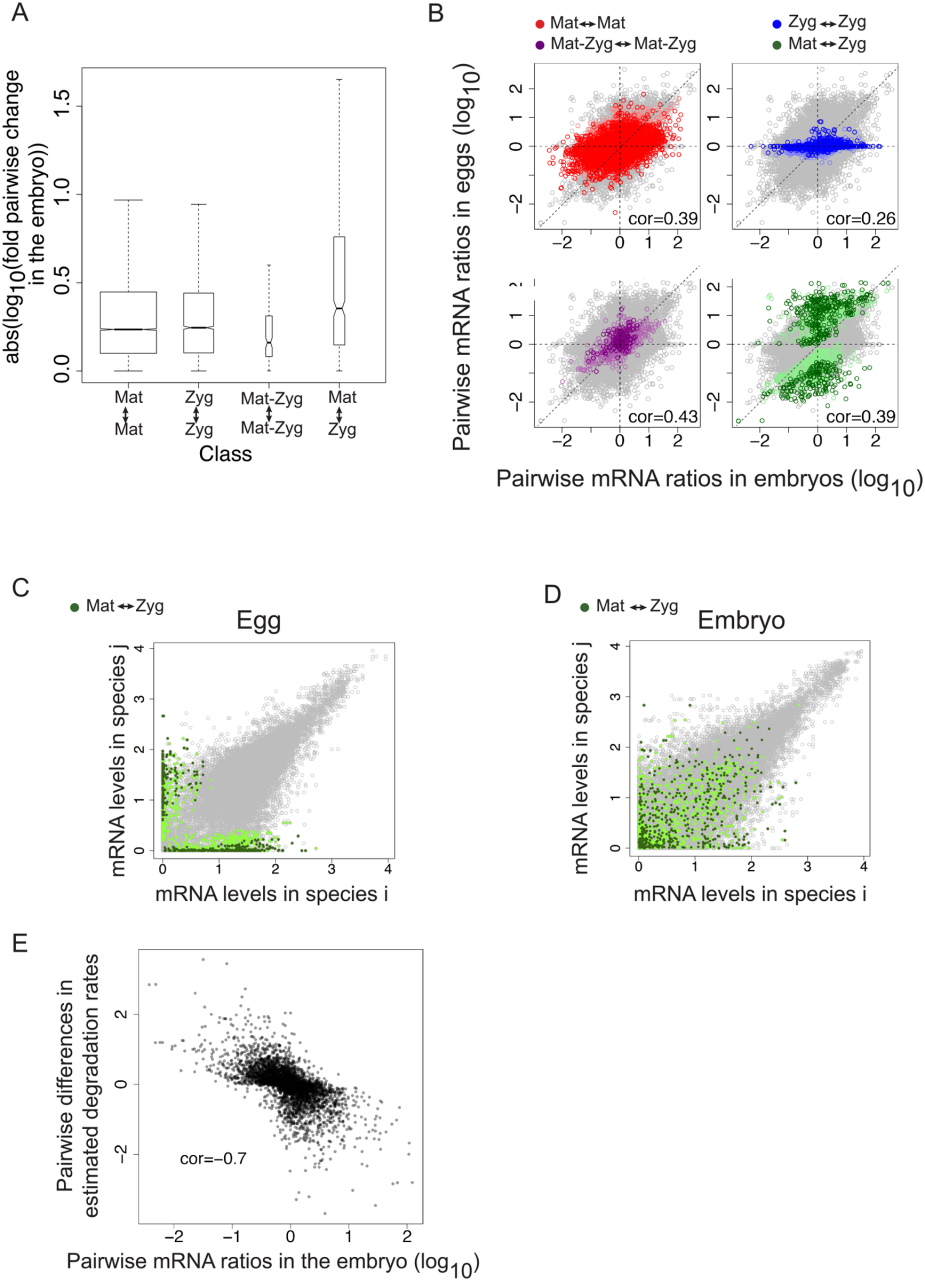
**A. Genes whose origin (“Mat <-> Zyg”) changes show higher species differences in embryonic mRNA levels compared to genes with conserved origin. B.** Divergence of mRNA levels between species is correlated with divergence of mRNA levels in the egg. We compared pairwise species differences of mRNA levels in the embryo and in the egg and found an overall positive correlation. We focused on the genes that had a conserved maternal (red), zygotic (blue) or Mat-Zyg (purple) origin in two species, or a change from maternal to zygotic (green). Grey points represent the complete dataset (all pairwise comparisons for the 5985 genes). **C/D.** Genes that have a divergent maternal or zygotic origin (“Mat <-> Zyg”) show highly divergent mRNA levels in the egg (**C**), which partially translates in the embryo (B). Points in green represent genes that are classified as maternal or zygotic in one species and have switched to zygotic or maternal in the other. Dark green was used for genes classified with high probabilities and light green was used for genes classified with low probabilities. **E.** Divergence of degradation rates correlates with embryonic expression in the embryo: an increase in mRNAs between species correlates with a decrease in degradation rates. We compared species differences in estimated degradation rates with species differences in mRNA levels in the embryo for the 667 genes that were predicted to have species-specific degradation rates according to a likelihood ratio test. Spearman correlation rho = 0.7, p. value < 2.2x10^-16^.

From the predictions of this model, we observe that most genes maintain their maternal or zygotic expression status across species. Genes that are maternal in one species almost always stay maternal in other species (Fig 5B). Similarly, genes that are categorized as having only zygotic expression at this stage primarily remain zygotic in other species (Fig 5B). Genes that remain in the same category (“Mat”, “Zyg”) across species are also more likely to retain similar transcript abundances across species than genes that switched from maternal to zygotic origin (or vice versa) (Fig 6A; Wilcoxon test p-value < 10^−16^).

Comparing species changes in transcript levels in blastoderm embryos and unfertilized eggs, we can explore the maternal contribution to the patterns we observe in the embryo. Changes in embryos correlate with changes in eggs for all genes (Spearman’s rho = 0.33), and notably for maternal genes (Spearman’s rho = 0.39 for genes categorized as maternal with high probability, red points labeled “Mat <-> Mat”, Fig 6B), indicating that the divergence in transcript level is partly maternal in origin. Changes in the embryo are unsurprisingly poorly predicted by changes in the egg for genes that stay zygotic across species (Fig 6B, blue dots). Changes for genes that stay “Mat-Zyg” across species have the highest correlation between embryos and unfertilized eggs (yellow points labeled “Mat-Zyg <-> Mat-Zyg”, Fig 6B, rho ~0.45, p-value < 2x10^-19^), suggesting that transcript levels may be under high levels of developmental constraint, as they manage to remain constant despite maternal degradation and zygotic transcription (although this category was poorly classified using the SVM). Genes that change from maternal deposition to zygotic transcription, and *vice versa*, across species harbor similar divergence of transcript abundances in unfertilized egg and embryo (Spearman’s rho = 0.39, green points labeled “Mat <-> Zyg” in Fig 6B). This latter correlation is probably driven by differences in what remains of maternal deposition in the embryo, although our dataset does not allow distinguishing this possibility. Interestingly, some of these genes that switch between maternal deposition and zygotic transcription have very different levels in the unfertilized egg (Fig 6C) but show much better conserved levels in the embryo (Fig 6D, green dots located along the diagonal). This suggests that despite a drastic change in maternal deposition in the egg, there has been conservation of mRNA levels for these genes in the embryo (and not in the egg).

Divergence of maternal mRNA deposition is a good but incomplete predictor of gene expression divergence in embryos (Fig 6B). Under the hypothesis that embryonic expression levels of maternal genes are solely dependent on the initial maternal deposition and subsequent degradation, our results suggest that degradation rates for maternal genes have diverged between *D*. *melanogaster*, *D*. *yakuba*, *D*. *pseudoobscura* and *D*. *virilis*. In order to test this hypothesis, we used the 1374 genes that are predicted as maternal with high probability in all four species (of note, this four-way conservation likely minimizes false positive "Mat" genes) and estimated their degradation rate according to the following formula, with r representing the degradation rate:

mRNA[embryo]~mRNA[egg](1-r)

mRNA[embryo] and mRNA[egg] were obtained from the different abundance measurements of the embryo and egg replicates, respectively. The degradation rate r was estimated as the value that minimizes “mRNA[embryo]—mRNA[egg](1-r)“. We compared two models in which the degradation rate was either the same between species or species-specific by comparing degradation rates estimated either on each species separately or on the four species together. For about half of the genes (667/1374), the model of a species-specific degradation rate was more likely according to a likelihood ratio test and the corresponding degradation rates showed at least a twofold difference between any two species (this proportion varies from a third to half depending on normalization). Accordingly divergence of embryonic mRNA levels for these genes correlates with differences in predicted degradation rates (Fig 6E): an increase in degradation rates results in a decrease in embryonic mRNA levels (Spearman correlation of ~0.7). These differences in degradation rates poorly correlate with differences in mRNA content of the unfertilized egg samples (Spearman correlation of ~-0.15, S9 Fig).

## Discussion

The analysis we report here is the first evolutionary study of gene expression during early *Drosophila* development using sex-specific embryonic transcriptome profiling of species spanning the genus *Drosophila*. At this time, the transcriptional environment of the blastoderm embryo is unique as compared to later stages of development for two reasons. First, while some embryos carry two X chromosomes and others only one, canonical MSL-mediated X chromosomal dosage compensation is not yet established [1,2,12–14]. Second this is the period when developmental control is being handed off between the maternal and zygotic genomes [3]. This is all taking place at the same time that the embryo is undergoing developmental processes (such as formation of body axes) that are known to be dependent on concentration of proteins encoded by transcripts, and are absolutely critical to the formation of the animal.

### Sex and the single embryo

The evolutionary processes that drive sex-biased gene expression are likely very different in adults as compared to early embryos. For this reason, it is interesting to compare the gene expression phenotypes in female and male embryos, relative to later developmental stages, to determine where the patterns of transcript abundance differ.

The most striking finding from our sex-specific data across species is the dominance of female-biased genes. This is in stark contrast to adults, where in almost all species, there are far more male-biased genes than female-biased genes [8]. While we observe female-biased genes to be more numerous, male-biased genes have a higher magnitude of sex-bias in embryos as well as in adults. These results are consistent with an incomplete X chromosomal dosage compensation, prior to the establishment of MSL-mediated dosage compensation. As we have shown in *D*. *melanogaster*, this incomplete dosage compensation causes large numbers of somewhat female-biased genes (generally, at the most 2x higher than male-biased genes), whereas the small number of genes we observe having male-bias are those whose transcript levels are being ramped up in order to establish MSL-mediated dosage compensation (such as the *msl* genes themselves), and thus are considerably more than 2x higher in males ([1], we observe this trend for *msl-2* in all four species).

We observe an overrepresentation of female-biased genes on the shared X chromosome (Müller A) in the embryo to be common in all species, as has been repeatedly found for later developmental stages in *Drosophila* [6,7,27,28]. However, unlike these earlier studies [6,7,27,28], we do not observe a significant deficiency of male-biased genes of the shared X chromosome across species in the embryo. This pattern may be influenced by the incomplete nature of early zygotic dosage compensation, the small total number of male-biased genes at this stage, or other differences between embryo and later developmental stages. However, lineage-specific patterns of sex-bias are far less clear. In *D*. *virilis*, lineage-specific female or male-biased genes show no enrichment or paucity on the X chromosome (Müller A) while male-biased genes are observed to be both under-represented (*D*. *melanogaster*) and over-represented (*D*. *yakuba*) on the X chromosome. The basis for this confusing pattern is likely due to a combination of many factors, including the high number of female-biased genes shared amongst all four species.

The dominant signal we observe for genes showing species-specific sex-bias is the female-bias of *D*. *pseudoobscura*, consistent with studies in other developmental stages[8,29]. In the lineage leading to this species, a chromosomal fusion event formed a two-armed X chromosome, consisting of both Müller A and Müller D, whereas the other species have only the acrocentric Müller A element as an X chromosome. *D*. *pseudoobscura* has a large number of female-biased genes on Müller D, which are not female-biased in any other species, likely due to this chromosome being X linked, and compounded by the incomplete nature of dosage compensation during this time in development. This *D*. *pseudoobscura-*specific increase in female-bias transcript level on Müller D is due to a decrease in transcript levels in males compared to other species, consistent with the number of Müller D templates available. What is more remarkable is that this evolution of sex-bias by a decrease of transcript levels in one sex (rather than an increase in the other sex) is generalizable. We observe this pattern for most genes with lineage specific sex-bias, in both females and males, in all species. This would suggest that there may be a larger number of evolutionary paths to decreasing expression level, or perhaps many genes in the early embryo are transcribed at close to their maximal expression level, so that increasing transcript level would be difficult.

### Lack of evidence for embryonic faster-X effect on transcript level

Another property of the embryonic transcriptome that may be quite distinct from the adult is the lack of a faster-X effect. Evolutionary theory holds that the X chromosome has the potential to play a disproportionately large role in divergence and speciation [30–32]. This increased level of divergence, termed the faster-X effect, has been found at the protein level by genomic studies, in some, but not all, lineages (see [31,33,34]). The faster-X effect has also been examined at the level of gene expression. Previous studies in *Drosophila* have demonstrated the higher divergence in gene expression on the X chromosome as compared to the autosomes[25,26]. The only other study to address this effect in embryonic gene expression did report higher divergence of transcript level on the X [26], but we did not replicate this result. Extensive probing into the dataset from this study and comparisons with ours (see results, Fig 4 and S3–S6 Figs), demonstrated that the differences in our findings were largely features of the data, rather than how it was processed or what subset of genes were compared. The primary difference in the methodology for gathering the samples was the use of pooled collection of embryos *vs*. our single embryo approach. In our data, we can only detect expression divergence on the X in the unfertilized eggs. It is likely that the pooled embryo samples used in [26] included some number of individuals from earlier stages (even eggs), which might explain at least to some extent why gene expression on the X chromosome appears more divergent in embryos collected in pools rather than as individuals.

Overall, our results demonstrate a faster-X effect for gene expression only in the earliest stages of embryogenesis. These stages of development are those dominated by maternally deposited transcripts, suggesting that the faster-X effect is observable in the mother rather than the embryo. Especially considering the high level of expression conservation between even distantly related species, the lack of a faster-X signal in our data may point to different selective pressures at this stage, such as high levels of developmental constraint on transcript levels in the early embryo.

### Maternal contribution to blastoderm expression divergence

The blastoderm embryo transcriptome consists of transcripts both maternally deposited and zygotically transcribed[1,35]. Thus, the variation between species in the transcripts present at this developmental stage may be either maternal or zygotic in origin. This leads in turn to a set of fundamental but largely unaddressed questions about the conservation of gene expression in the mother or the zygote. For example, does the origin of a gene in a species (maternal or zygotic) inform about the origin of this gene in another species? Our model suggests that this is the case: a maternal gene in one species is likely to be a maternal gene in other species, and a gene transcribed zygotically in one species is likely to also be zygotically transcribed in other species. Unsurprisingly for maternal genes, differences of transcript level in the blastoderm embryo were already established in the egg and are thus maternal in origin. We do find that a small number of genes are likely to be maternal in one species and zygotic in another, or *vice versa*, and these genes are more divergent in their transcript level between species in the blastoderm embryo.

Cellular transcript content is the result of both transcript production and transcript degradation. The contribution of transcript degradation to transcript level “steady state” has been modeled in model systems [36–40], but has largely been neglected as a source of divergence between species. However, most studies have interpreted divergence of mRNA levels between species as divergence of transcription, neglecting the contribution of mRNA degradation (with the noticeable exception of yeast [41]). The earliest stages of development before zygotic transcription starts are the only time in the life of an animal when one can study mRNA degradation exclusively, because transcripts of maternal genes are degraded from the maternally deposited pool and not transcribed by the zygote yet. We used this property to estimate and compare divergence of degradation rates for maternal genes between species. We find evidence for general species-specific differences in modeled transcript degradation rates, which largely contributes to the difference in maternal transcript levels at the blastoderm stage. The functional consequences of these inferred changes in degradation rates remain unexplored.

### Advantages of single embryo approaches

A number of the findings reported here were only made possible due to the use of single embryos in experiments. The sex-specific embryo data would have been considerably more difficult to collect by other methods. As we demonstrate here, this methodology has been especially fruitful when applied in comparative studies. The species we used here have different development times, and a tendency to lay different proportions of non-developing eggs. Collections of pools of embryos necessarily contain a distribution of developmental ages, and potentially can be “contaminated” by earlier or later stage embryos than the desired distribution. Both the stage distributions and levels of contamination are likely to vary between species. When different species contribute different numbers of non-developing eggs (often unfertilized eggs) to pooled samples, this adds considerable signal to inter-species comparisons (see Fig 1D), and confounds development differences with species differences. The ability to select individual embryos that have developed to the same (morphological) stage greatly improves accuracy and decreases development bias, which is critical in these cross-species studies.

## Methods

### Sample collection, library construction, sequencing

For single embryo collection, eggs were collected from 3–5 day old females of *D*. *melanogaster* (Oregon-R), *D*. *yakuba* (Tai18E2), *D*. *pseudoobscura* (MV2-25), and *D*. *virilis* (V46), dechorionated, and imaged under halocarbon oil to determine stage. Embryos were collected from a large number of mothers, so it is unlikely that multiple samples came from the same mother. Embryos were viewed on a Nikon Eclipse 80i light microscope, and were selected based on having completed cellularization but not yet having gastrulated. Embryos were then removed from the slide with a brush, cleaned of excess oil, placed into a drop of Trizol reagent (Ambion), and ruptured with a needle, then moved to a tube with more Trizol to be frozen at -80°C until extraction. RNA and DNA were extracted as in the manufacturer’s protocol, with the exception of extracting in an excess of reagent (1 mL was used) compared to expected mRNA and DNA concentration (see [1,2]).

Extracted DNA was subjected to whole genome amplification (Illustra GenomiPhi V2, GE Healthcare), and used for PCR assays to determine embryo sex. Primers were designed separately in each species, reactions multiplexed sets of primers to two different locations on the Y chromosome and a control locus not on the Y. Primer sets used can be found in S7 Table.

Extracted total RNA from single embryos was treated with TurboDNase (Ambion) prior to library construction. Embryo mRNA-Seq libraries were made for 3 replicate individual female embryos and three replicate male individuals per species. Egg mRNA-Seq libraries were constructed for three replicate individual eggs per species. mRNA-Seq libraries were constructed using TruSeq RNA sample preparation kits (Illumina), using standard protocols, and indexed to pool 12 samples (embryos or eggs) per lane. Library concentration was measured using the Qubit fluorometer (Life Technologies) and the qPCR-based Library Quantification kits (KAPA biosystems), and size was measured using the Bioanalyzer (Agilent). Libraries were sequenced on an Illumina HiSeq 2000 DNA Sequencer. Some samples were removed *a posteriori* because of lower quality (see Results) and we ended up using a total of 29 samples (22 embryo samples + 7 egg samples, listed in S8 Table).

### Data availability

All reads are available at the NCBI GEO (accession number of the project: GSE68062; accession number of each sample listed in S8 Table). mRNA-seq reads on the pool datasets had previously been deposited in the NCBI GEO (accession numbers GSM1228784 and GSM1228785).

### Data processing

We used the annotation version 5.39 for *D*. *melanogaster* and the annotation previously published for *D*. *yakuba*, *D*. *pseudoobscura*, and *D*. *virilis* [19].

We trimmed all sequenced tags so that their average quality was above 30 and mapped the tags to the transcripts using Bowtie2 [20] with command-line options ‘-a -N 1‘, thereby allowing all possible mappings. We then estimated transcript levels using eXpress [21]. Gene levels were obtained by summing levels over all transcripts of a gene. Gene levels were then normalized (see below), then log transformed (log10(normalized FPKM+1)). Orthology was assessed from [19].

Six normalization procedures were tested: (i) either no further normalization after eXpress, normalization after eXpress to the median (ii), to the 75^th^ percentile (iii), to the 95^th^ percentile (iv), TMM normalization (v) or quantile normalization (see [42] for review). The analyses presented in the main text were conducted on the 75^th^ percentile normalization but the different normalizations essentially gave similar results. A few representative plots similar to the main figures are given in S10 and S11 Figs for the 6 normalizations. In addition, tables created from the different normalized datasets are available on GEO (accession number GSE68062).

### Analysis of sex-bias

All data analysis was performed in R [43].

For Fig 2, sex ratios were calculated on non-log transformed expression levels. Genes were ranked according to the absolute log_2_(female:male) ratio, and the chromosomal distribution of the top 200 genes was assessed (Fig 2C).

For Fig 3, genes were ranked based on their female to male ratio (sex ratio). To avoid large sex ratios due to variance in transcript level for low abundance transcripts, we tested different transcript level cutoffs, and found that requiring the transcript be present in all four species at >0.33 FPKM on a log2 scale was sufficient. We define genes as being female-biased if their sex ratio was ranked in the top 20% (for other bias cutoffs see S1–S6 Tables) and as male-biased if their ratio was in the bottom 20%. Genes were defined as unbiased if their ratio fell within 10% of the median (i.e. the 40%-60% quantiles; see S1–S6 Tables for other cutoffs). Expected chromosomal distributions of genes for chi-squared tests were determined by the total number of genes identified as sex-biased, distributed based on the number of genes on each chromosome.

### Measurements of gene-wise phylogenetic variance to test the “faster X” hypothesis

Gene-specific variance of expression was reconstructed according to a Brownian motion model [44]. Under a Brownian motion model, continuous characters evolve randomly along a phylogeny following a random walk. We used the R package ape (function ‘‘ace”) with the following parameters: model = "BM", method = "REML", type = "continuous" [45].

### Comparison of chromosome-wise divergence of transcript levels between species

We approximated divergence of chromosome expression between species according to the correlation of gene expression for each chromosome. Per chromosome we measured Spearman correlation of transcript levels for all pairwise species combinations. We also generated 10,000 bootstrap replicates per chromosome of the pairwise Spearman correlations using the R function boot from the package boot.

We compared our dataset to the dataset from [23], which was obtained from the ArrayExpress database, using the accession number E-MTAB-404 [23,26]. Using this dataset that covers eight time windows during embryonic development, evidence for a faster X effect had been reported. We first verified that we indeed recovered the “faster X” effect in the microarray dataset [23] using our analysis pipeline. We found that expression was indeed more divergent on the X compared to other chromosomes, as published. We also noticed that the effect was most pronounced for early time points (S4 Fig).

Inter-species comparisons are done on datasets of orthologous genes. The method for pairing genes between species was done in very different ways in our study *vs* [23] (genome alignment in our case *vs* blast of microarray probes in the analysis by [23]). We thus checked whether differences between our dataset and the microarray dataset stems from differences in orthology assignment. We reassigned gene orthology in the [23] dataset by first identifying through BLAST which of our gene sequences matched the probes from the *D*. *pseudoobscura* and *D*. *virilis* microarrays. We removed any gene for which all probes assigned to one gene did not match the same gene from our annotations. Between species correspondence was based on [19]. We ended up with 1404 genes and then processed this dataset similarly to ours. We found that expression was still more divergent on the X chromosome after recalling orthologous genes in the microarray dataset (S5A Fig). Again, the effect was more pronounced for early time points.

Finally we found a correlation between overall chromosomal expression levels and expression divergence: the higher the expression in a chromosome, the higher the correlation between species (S6A Fig). The X chromosome systematically has a lower expression compared to the other chromosomes. This effect is much weaker with our dataset (S6B Fig). Overall however correlations between levels of expression and pairwise correlations between species on one hand and egg contamination in embryo pools on the other hand likely account for some but not all the discrepancy between both datasets.

### SVM classification of gene origin

In order to predict the gene class (maternal, zygotic of maternal+zygotic) based on egg and embryonic expression, we learned an SVM using the *D*. *melanogaster* 5408 genes for which a maternal / zygotic / maternal+zygotic classification had previously been established by [1] using the function “svm” of the R package e1071[46] using the parameters kernel =“radial”, method =“C-classification”, gamma = 0.01, cost = 10, and otherwise default parameters. To select those parameters, the SVM was learned on a training set of 2/3 of the dataset (3606/5408) and tested on the remaining 1/3 (1802/5408). 100 replicates (with 100 different training and test sets) were performed and the parameters were selected to maximize the number of correctly classified genes, in particular to minimize the number of maternal genes predicted as zygotic (or *vice versa*). Of note, among the variety of parameters that we tested, the kernel and cost values showed the most variation in accuracy while other parameters were very robust. An example of the SVM performance using the chosen parameters is given S7 Fig. Once the parameters were selected, we relearned the SVM on the complete *D*. *melanogaster* dataset of 5408 genes and applied it to the other species. Genes were further categorized depending on the confidence of the classification: the classification was considered “highly confident” if the associated probability was above 0.8, 0.7 and 0.8 for maternal, zygotic and Mat-Zyg genes, respectively. As described in the result section and shown in S7 Fig, the resulting classification of maternal and zygotic genes was very accurate whereas classification of Mat-zyg genes was of poor quality.

### Analysis of maternal contribution to blastoderm expression divergence

Degradation rates were estimated on a set of 1374 genes that had been classified as maternal with high probability in all four species according to the formula:

mRNA[embryo] ~ mRNA[egg] * (1−r) + epsilon

r being the degradation rate between eggs and embryos and epsilon following a normal distribution of mean 0 and variance the average variance of the dataset values. Non-log-transformed values were used for this analysis. All replicates were used (not the averaged values). r was estimated using the R function “optimize” as the value that minimizes “mRNA[embryo]—mRNA[egg](1-r)“. Two models were used and compared to optimize r for each gene: either r was common among the species, or it was species-specific. In other words, the optimization was either done on each species separately (species-specific degradation rate) or on the whole dataset together (species-common rate). The likelihood of both models was then calculated gene-wise using the above formula and the optimized r. Genes with species-specific degradation rates were selected according to a Likelihood ratio test with four degrees of freedom (Benjamini & Hochberg corrected p-value < 0.001) The list of genes with species-specific degradation rates was further refined by imposing a fold change > 2 between any two species.

## Supporting Information

S1 Fig Gene expression divergence follows phylogenetic distance. We approximated gene expression divergence in eggs and embryos as 1 –Spearman correlation and then compared it to species evolutionary distance, as number of substitutions per base[47].
(PDF)

S2 Fig Highly expressed genes exhibit a low degree of sex-bias. Gene-wise female:male sex ratio were compared to average levels of expression in *D*.*melanogaster*, *D*.*yakuba*, *D*.*pseudoobscura* and *D*.*virilis* blastoderm embryos.
(PDF)

S3 Fig Bootstrapped distributions of Spearman's rank correlation across chromosomes in males, females and unfertilized eggs for the six pair-wise species comparisons between *D*. *melanogaster*, *D*. *yakuba*, *D*. *pseudoobscura* and *D*. *virilis*. Dashed line represents the Spearman’s correlation for genes on Müller element A (X chromosome). 10,000 bootstrap replicates were computed per comparison.
(PDF)

S4 Fig Bootstrapped distributions of Spearman rank correlation across chromosomes in embryos for the 15 pair-wise species comparisons between six species along eight time points from the microarray study published by [23]. Dashed line represents the Spearman’s correlation for genes on the Müller element A (X chromosome). Orthology was obtained from [23].
(PDF)

S5 Fig **A. Bootstrapped distributions of Spearman rank correlation across chromosomes in males, females, eggs and three representative time points from a dataset previously published by [23].** This dataset consists of pools of embryos collected during eight developmental time windows. The analysis was based on the 1404 genes for which we could assign clear orthology from the microarray probe sequences in *D*. *pseudoobscura* and *D*. *virilis* [23]. Dashed line represents the Spearman’s correlation for genes on Müller element A. **B.** Spearman rank correlations between embryos or eggs (this dataset) and pools of embryos from the microarray dataset for the three species common in both studies: *D*. *melanogaster*, *D*. *pseudoobscura* and *D*. *virilis*. Time point 3 correlates the best with our embryo samples whereas time point 1 is very similar to eggs, which probably reflects a larger proportion of maternally deposited transcripts harvested from early time points. As observed with our pools of embryos (Fig 1D), the proportion of the egg component varies between species.
(PDF)

S6 Fig Spearman’s rank correlation across chromosomes is correlated with average expression levels for most pairwise comparisons from [23]. Spearman’s correlations across chromosomes were compared with average expression levels for all possible pairwise comparisons in [23] dataset (**A**) or ours (**B**). Expression on Müller element A is systematically lower than in other chromosomes in both males, females, eggs and pools of embryos. However only in (**A**) are Spearman’s correlations and levels of expression correlated.
(PDF)

S7 Fig The SVM was learned on 2/3 of the *D*. *melanogaster* and tested on the last 1/3 if the dataset. We compared the known classification of genes with the SVM output. Most genes maternal were predicted as maternal and almost all zygotic were predicted as zygotic. On the opposite, prediction of maternal-zygotic genes was much less successful, potentially because this group is less homogeneous.
(PDF)

S8 Fig SVM classification of the origin of an mRNA transcript in the embryo. mRNAs in the blastoderm embryo can have two possible origins: they were either maternally deposited into the egg and persisted until this stage, or they were actively transcribed in the embryo. This means that genes can be classified in three categories: purely maternal genes (called “Mat”), purely zygotic genes (called “Zyg”), or genes for which some molecules were deposited and some were zygotically transcribed (called “Mat-Zyg”). This classification has been previously established in *D*. *melanogaster* [1]. mRNA levels tend to partition between the different classifications on a 2D plot comparing eggs and embryos. We learned an SVM on the *D*. *melanogaster* dataset and used it to predict gene classification in the other species. Each classification was associated a confidence value, as “high” or “low”. This figure is an extended version of Fig 5A.
(PDF)

S9 Fig Divergence of degradation rates poorly correlates with expression in the egg. We compared species differences in estimated degradation rates with species differences in mRNA levels in the egg for the 667 genes that were predicted to have species-specific degradation rates according to a likelihood ratio test. Spearman correlation rho = -0.15, p. value < 2.2x10^-16^). In comparison, Spearman correlation was 0.7 for embryo samples (Fig 6E).
(PDF)

S10 Fig Comparison of the effect of dataset normalization on the analysis (I). A few representative plots of the different analyses described in this manuscript are shown for all 6 normalizations tested. **A.** Variation in the size of the dataset, depending on normalization. p75 normalization, that was used for the main figures, is highlighted in red. **B.** COA analysis as described Fig 1D. **C.** Chromosomal distribution of sex biased genes, as described in Fig 2C. **D.** The increase in transcript level sex-ratio on Müller element D in *D*. *pseudoobscura* is due to a decrease in male transcript abundances specifically in *D*. *pseudoobscura*, as described in Fig 2D.
(PDF)

S11 Fig Comparison of the effect of dataset normalization on the analysis (II). A few representative plots of the different analyses described in this manuscript are shown for all 6 normalizations tested. **A.** Chromosomal distribution of inter-species gene expression divergence in females, males and eggs, as in Fig 4. **B.** Divergence of degradation rates correlates with embryonic expression in the embryo, as described in Fig 6E.
(PDF)

S1 Table Numbers of sex-biased genes and magnitude of log_2_ sex ratio in the sex-biased genes for each species. The p-value given is the Wilcoxon test for a difference in median between the female and male sex-biased genes at a given cutoff of sex-bias (female to male ratio).
(XLSX)

S2 Table Chromosomal distribution of genes that are sex-biased in all 4 species. At different quantile cutoffs for sex-bias, we report number of genes that are sex-biased in all four species on each chromosome arm. We calculate the expected chromosomal distribution based on the observed total number of sex-biased genes and the relative number of genes on each chromosomal arm. We report the p-value from a chi-squared test for the difference between the observed and expected number.
(XLSX)

S3 Table Chromosomal distribution of genes showing no sex-bias in all four species. At different quantile cutoffs for not being sex-biased (unbiased), we report number of genes that are unbiased in all four species on each chromosome arm. We calculate the expected chromosomal distribution based on the observed total number of unbiased genes and the relative number of genes on each chromosomal arm. We report the p-value from a chi-squared test for the difference between the observed and expected number.
(XLSX)

S4 Table Numbers of genes that are sex-biased only in a single species. At different quantile cutoffs for sex-bias and non-bias, we report number of genes that are sex-biased in only one species, and unbiased in the other three species.
(XLSX)

S5 Table Chromosomal distribution of genes that are sex-biased in only one species. At different quantile cutoffs for sex-bias and non-bias, we report number of genes that are sex-biased in only one species, and unbiased in the other three species, on each chromosome arm. We calculate the expected chromosomal distribution based on the observed total number of sex-biased genes and the relative number of genes on each chromosomal arm. We report the p-value from a chi-squared test for the difference between the observed and expected number.
(XLSX)

S6 Table For genes that are sex-biased in only one species, we look at whether the sex-bias is due to increase in expression in females or males. For example, for a female-biased gene in species 1, we ask the proportion of these genes where female expression is higher in species one than species 2, and the proportion of these genes where the male expression is higher in species 1 than species 2. This address the question as to whether the evolution of female-biased genes comes on average from an increase in female expression, or a decrease in male expression. We calculate the significance of the bias, using the null that the proportion should be 50/50 as an genes is equally likely to become sex-biased by a change in female or male expression. The p-value is calculated using a binomial sign test of this null hypothesis.
(XLSX)

S7 Table Primer mixes used for PCR genotyping for each species. For each species, primers were constructed to two different loci on the Y chromosome (Y), and one locus not on the X chromosome (control). The Y chromosomal primers for D. pseudoobscura were taken from Carvalho and Clark, 2005 [48].
(XLSX)

S8 Table Description of the 29 samples sequenced and analyzed in this study. Name of the sample, species, sex, developmental stage as well as GEO accession number of the sequencing output are listed. Two additional samples (pse.F.3 and vir.M.1) that had originally been sequenced but subsequently removed from the analysis are also shown in grey.
(XLSX)
