# Supplementary figures and images for: Sex Bias and Maternal Contribution to Gene Expression Divergence in Drosophila Blastoderm Embryos
Source: PLoS Genet. 2015 Oct 20;11(10):e1005592. doi: 10.1371/journal.pgen.1005592 (PMC4618353; doi:10.1371/journal.pgen.1005592)

Figure S1

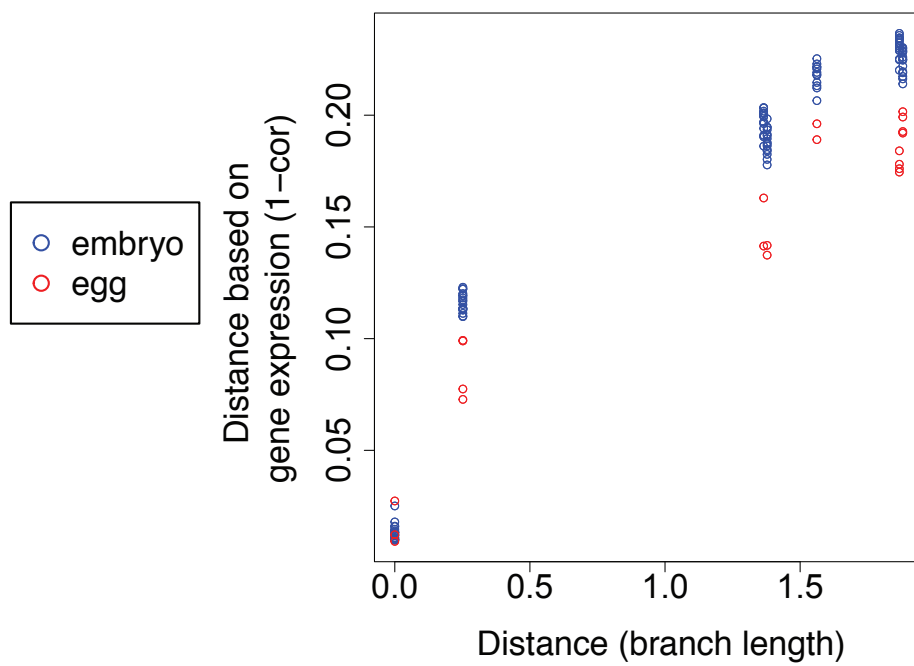

Supplement: S1 Fig — We approximated gene expression divergence in eggs and embryos as 1 –Spearman correlation and then compared it to species evolutionary distance, as number of substitutions per base[47]. (PDF) [file pgen.1005592.s001.pdf]

Figure S2

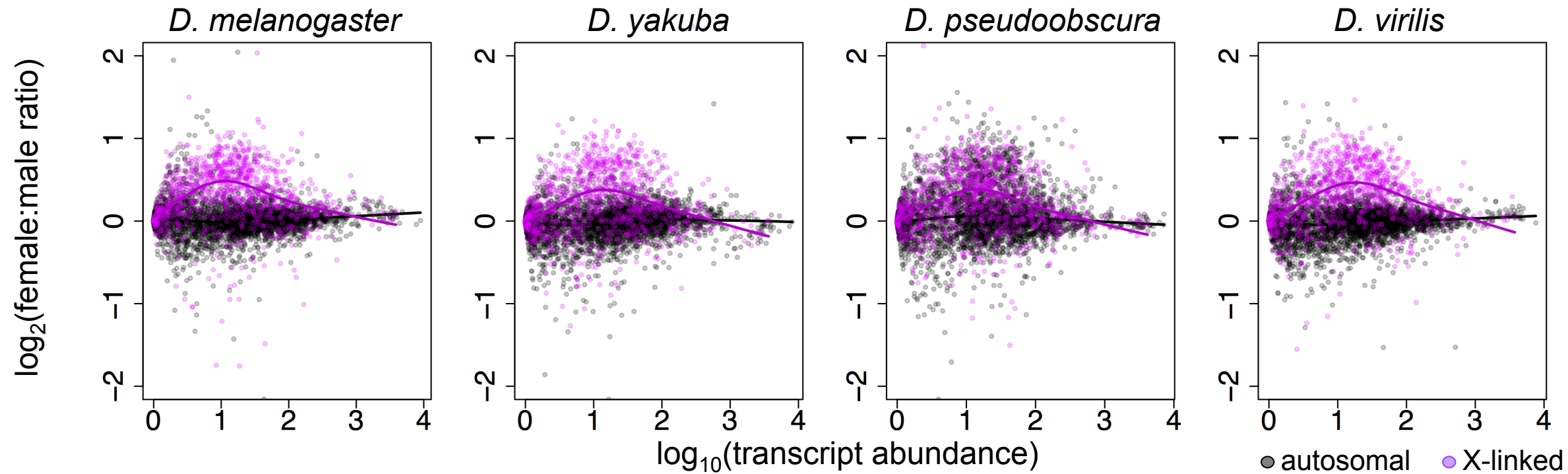

Supplement: S2 Fig — Gene-wise female:male sex ratio were compared to average levels of expression in D.melanogaster, D.yakuba, D.pseudoobscura and D.virilis blastoderm embryos. (PDF) [file pgen.1005592.s002.pdf]

Figure S3

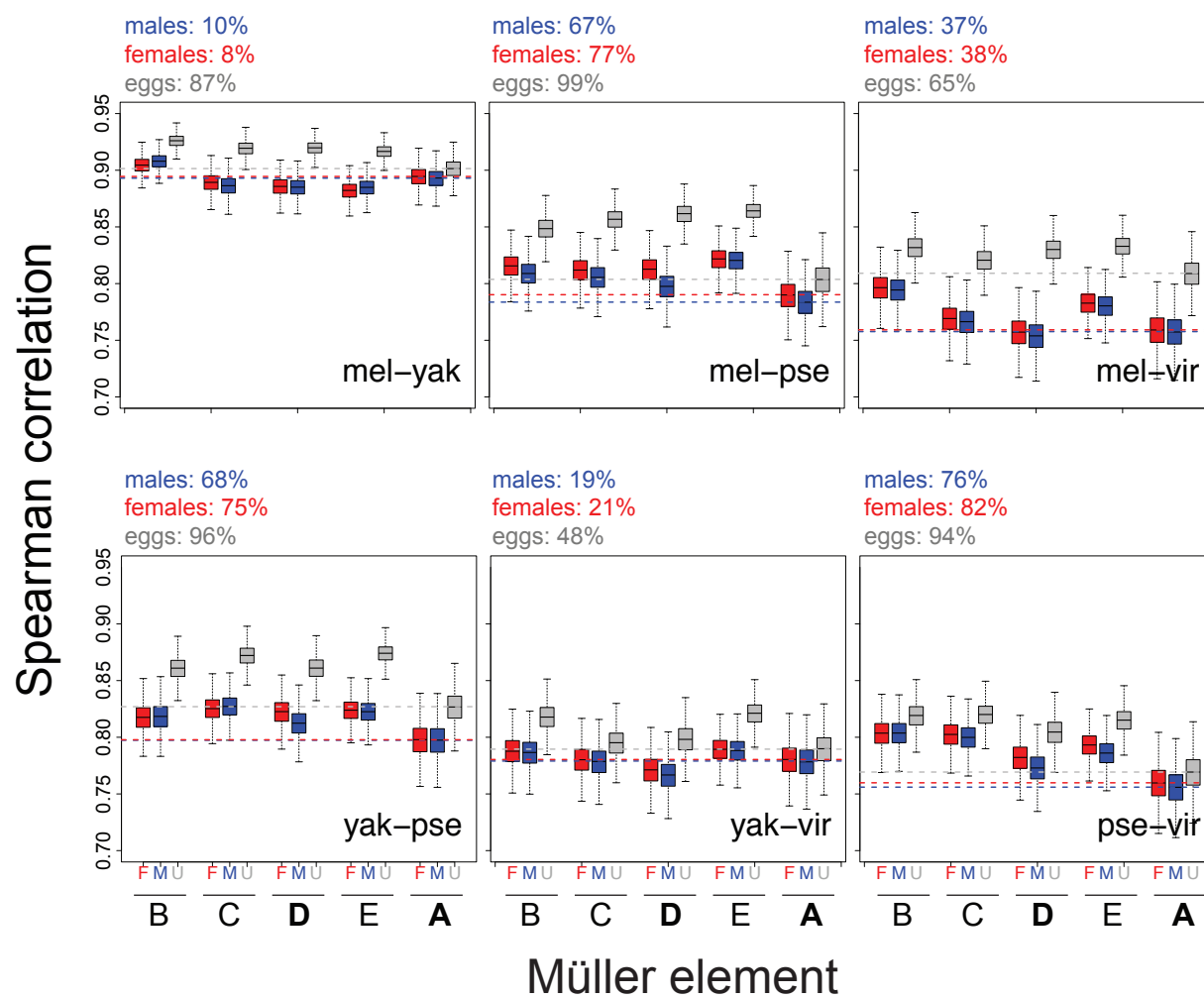

Supplement: S3 Fig — Dashed line represents the Spearman’s correlation for genes on Müller element A (X chromosome). 10,000 bootstrap replicates were computed per comparison. (PDF) [file pgen.1005592.s003.pdf]

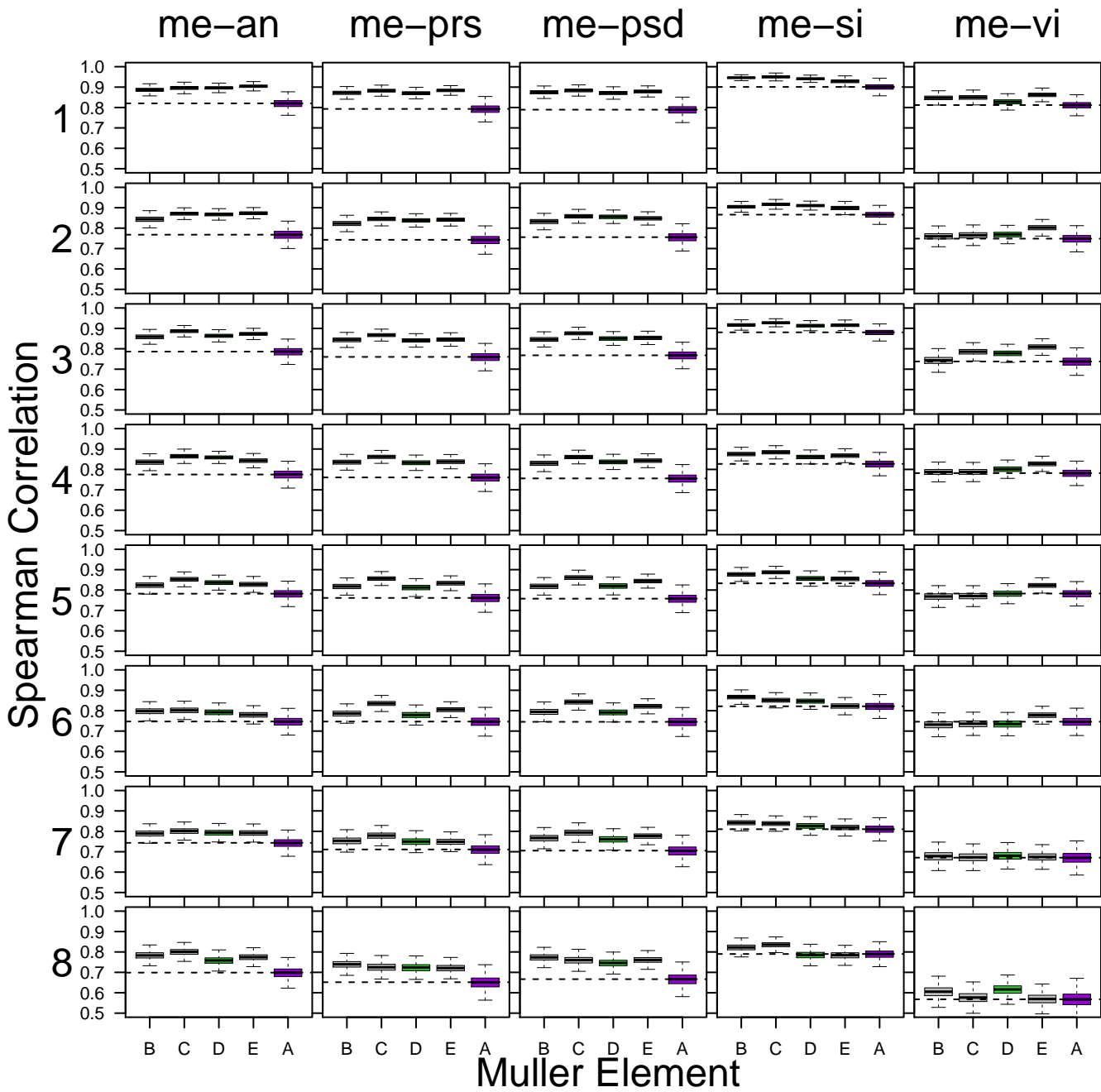

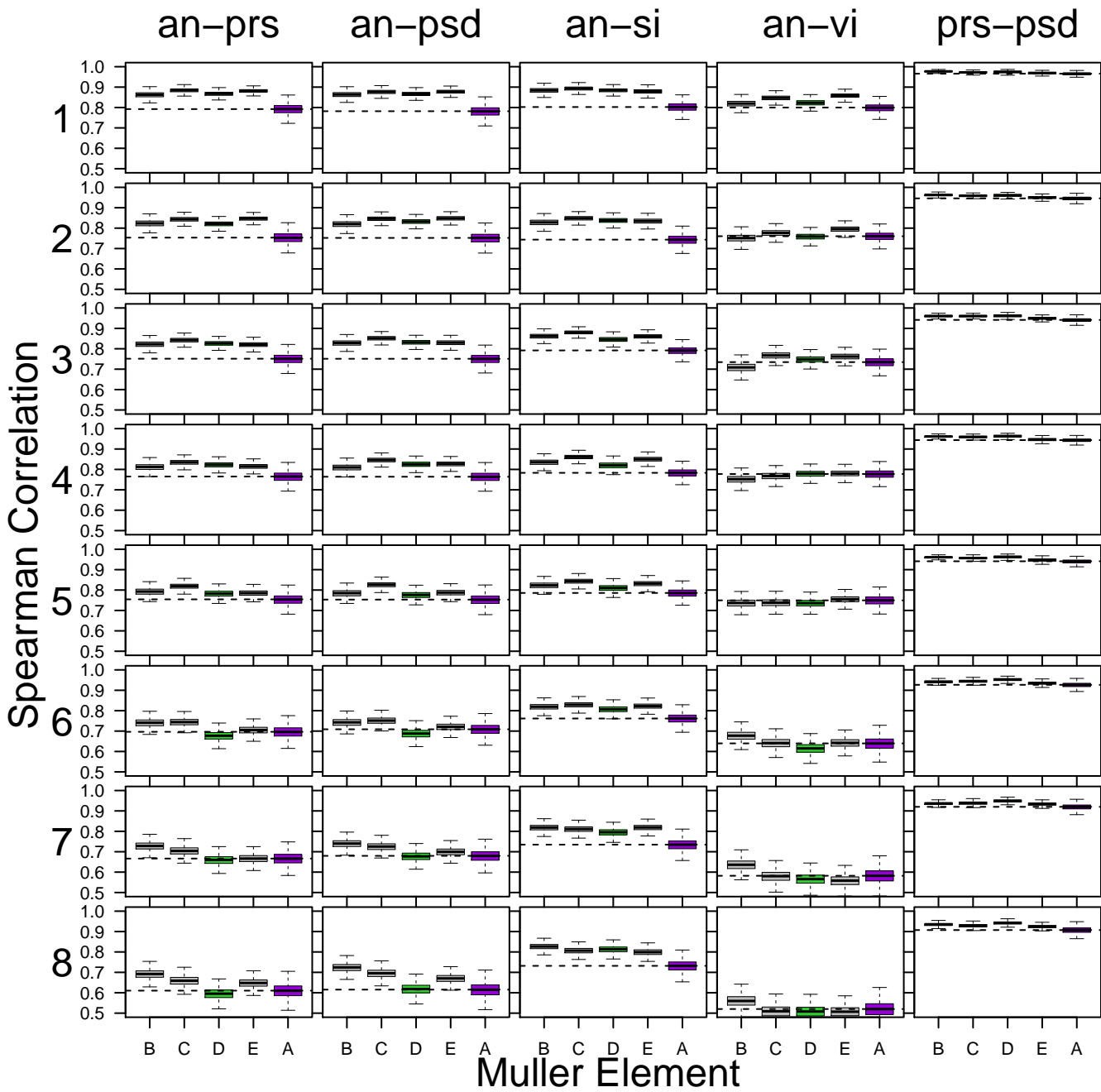

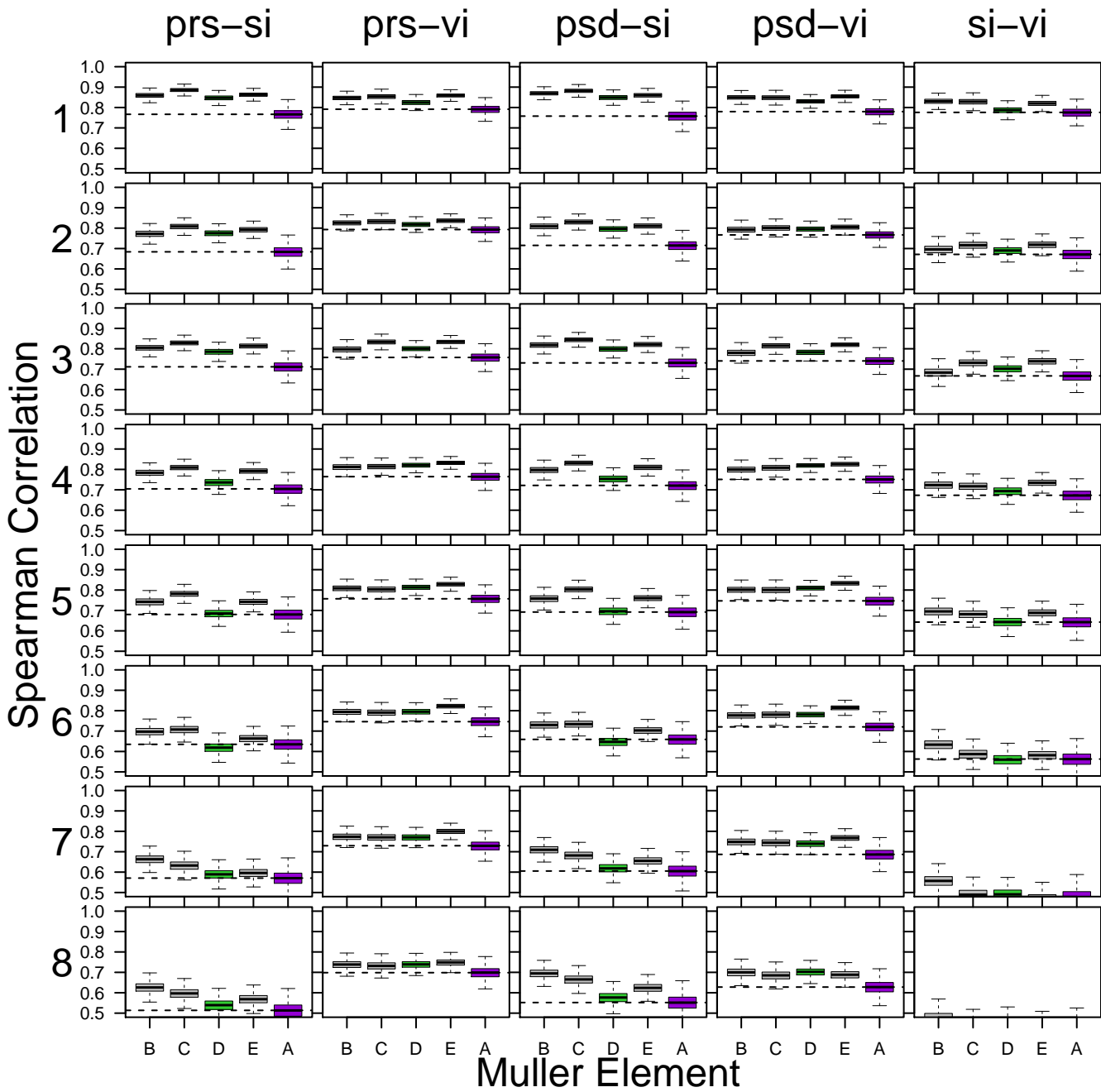

Supplement: S4 Fig — Dashed line represents the Spearman’s correlation for genes on the Müller element A (X chromosome). Orthology was obtained from [23]. (PDF) [file pgen.1005592.s004.pdf]

A

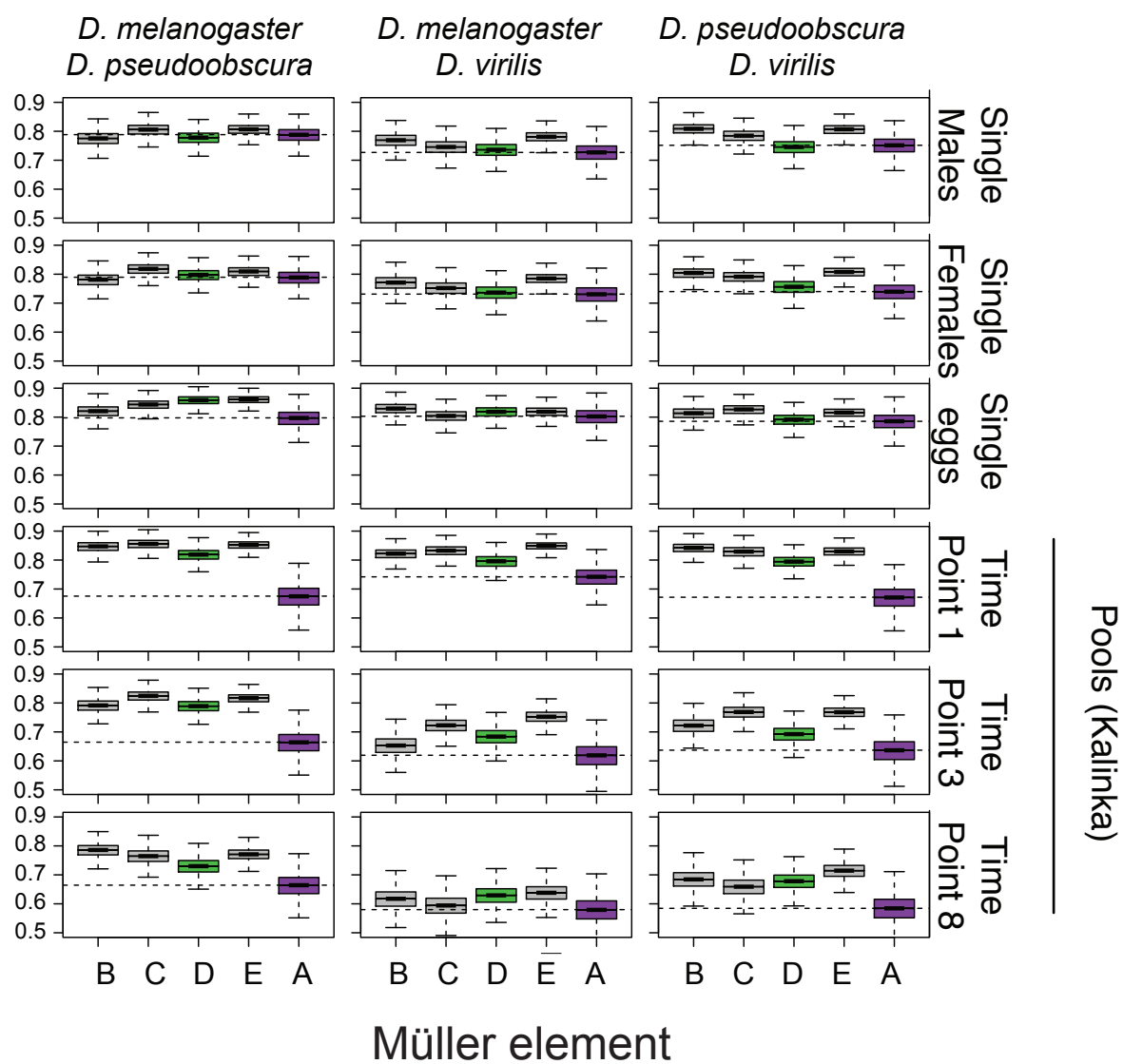

B

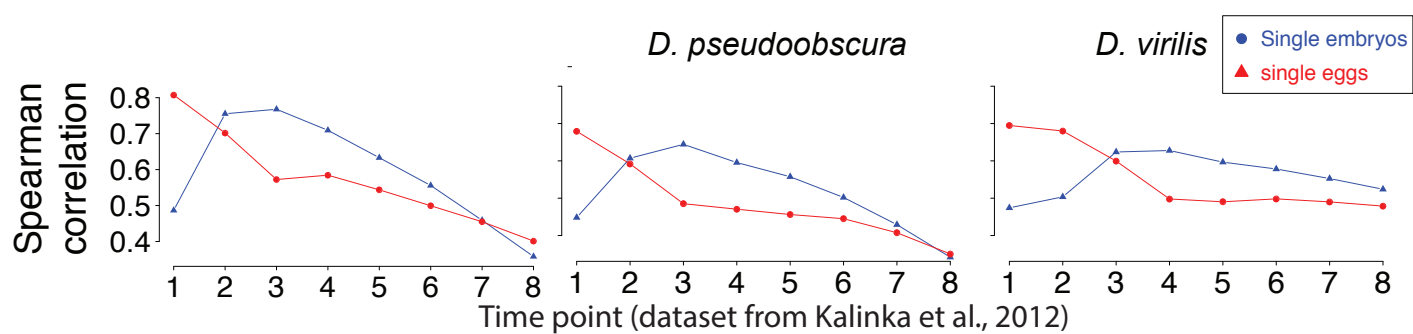

Supplement: S5 Fig — A. Bootstrapped distributions of Spearman rank correlation across chromosomes in males, females, eggs and three representative time points from a dataset previously published by [23]. This dataset consists of pools of embryos collected during eight developmental time windows. The analysis was based on the 1404 genes for which we could assign clear orthology from the microarray probe sequences in D. pseudoobscura and D. virilis [23]. Dashed line represents the Spearman’s correlation for genes on Müller element A. B. Spearman rank correlations between embryos or eggs (this dataset) and pools of embryos from the microarray dataset for the three species common in both studies: D. melanogaster, D. pseudoobscura and D. virilis. Time point 3 correlates the best with our embryo samples whereas time point 1 is very similar to eggs, which probably reflects a larger proportion of maternally deposited transcripts harvested from early time points. As observed with our pools of embryos (Fig 1D), the proportion of the egg component varies between species. (PDF) [file pgen.1005592.s005.pdf]

A

Kalinka et al. (time point 3)

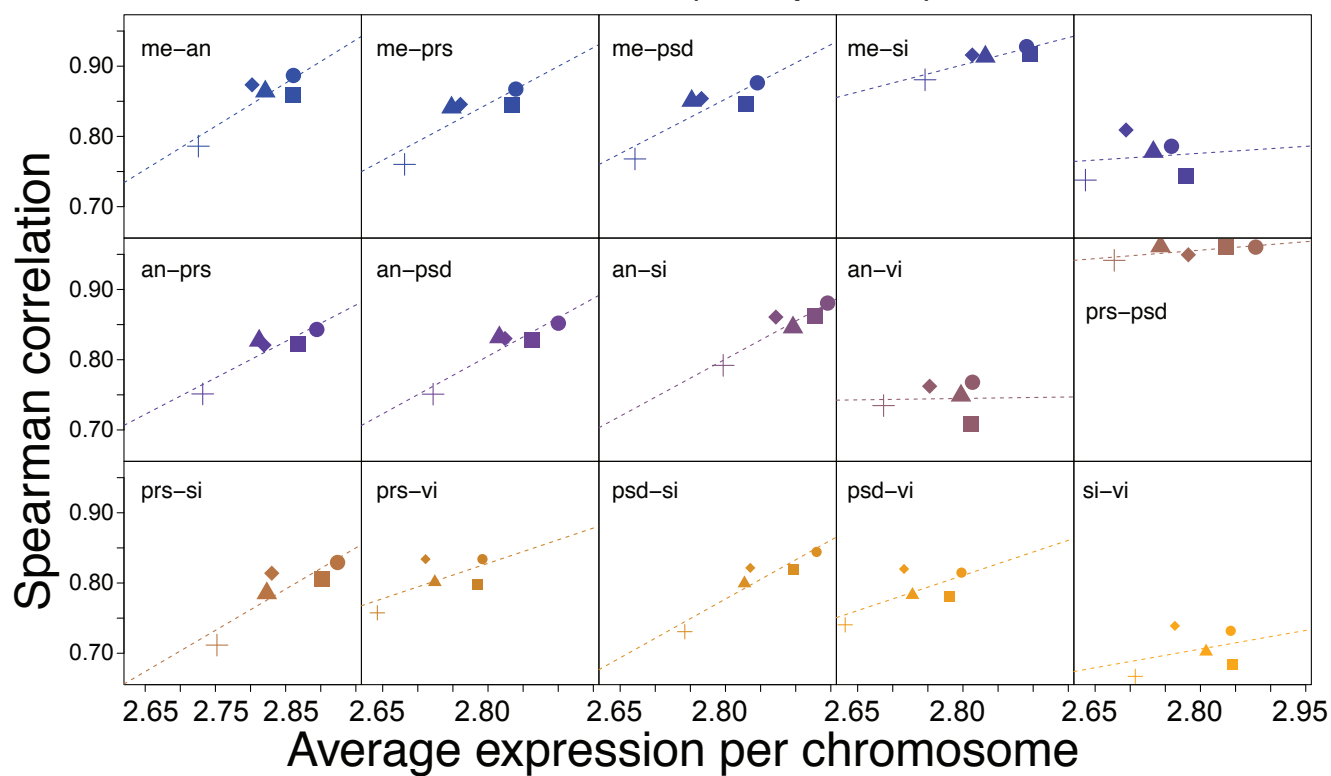

B

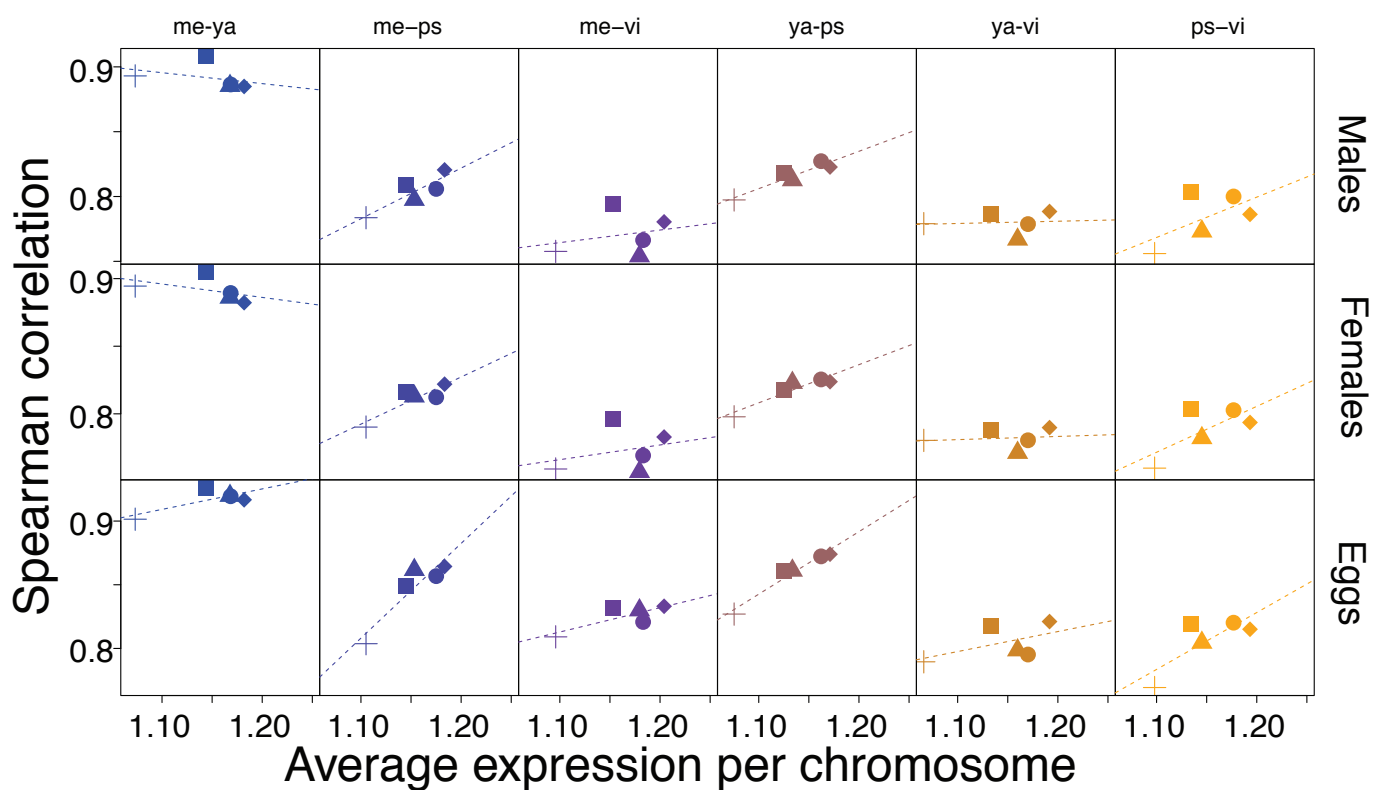

Supplement: S6 Fig — Spearman’s correlations across chromosomes were compared with average expression levels for all possible pairwise comparisons in [23] dataset (A) or ours (B). Expression on Müller element A is systematically lower than in other chromosomes in both males, females, eggs and pools of embryos. However only in (A) are Spearman’s correlations and levels of expression correlated. (PDF) [file pgen.1005592.s006.pdf]

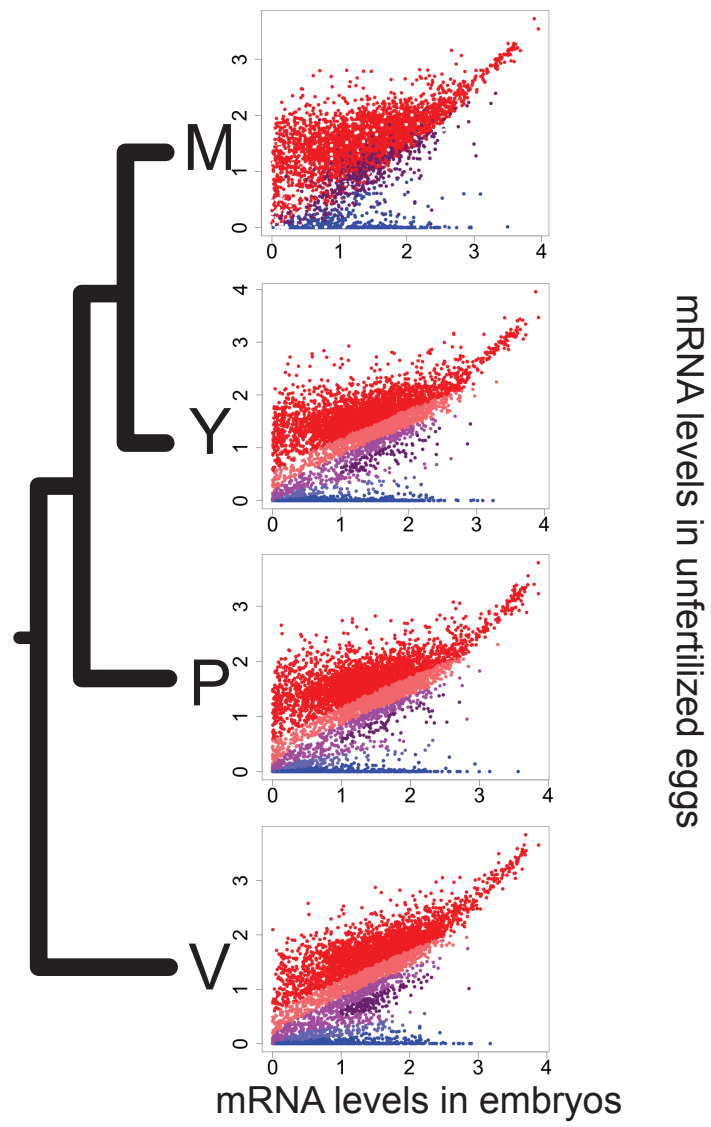

Supplement: S8 Fig — mRNAs in the blastoderm embryo can have two possible origins: they were either maternally deposited into the egg and persisted until this stage, or they were actively transcribed in the embryo. This means that genes can be classified in three categories: purely maternal genes (called “Mat”), purely zygotic genes (called “Zyg”), or genes for which some molecules were deposited and some were zygotically transcribed (called “Mat-Zyg”). This classification has been previously established in D. melanogaster [1]. mRNA levels tend to partition between the different classifications on a 2D plot comparing eggs and embryos. We learned an SVM on the D. melanogaster dataset and used it to predict gene classification in the other species. Each classification was associated a confidence value, as “high” or “low”. This figure is an extended version of Fig 5A. (PDF) [file pgen.1005592.s008.pdf]

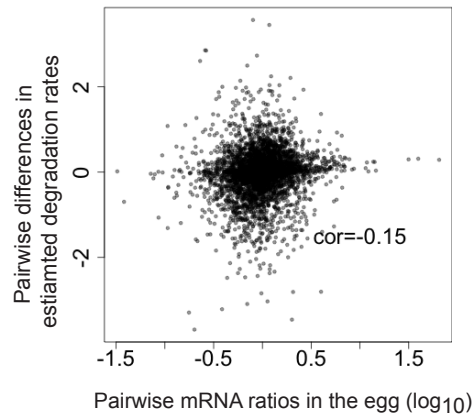

Supplement: S9 Fig — We compared species differences in estimated degradation rates with species differences in mRNA levels in the egg for the 667 genes that were predicted to have species-specific degradation rates according to a likelihood ratio test. Spearman correlation rho = -0.15, p. value < 2.2x10^-16^). In comparison, Spearman correlation was 0.7 for embryo samples (Fig 6E). (PDF) [file pgen.1005592.s009.pdf]

A

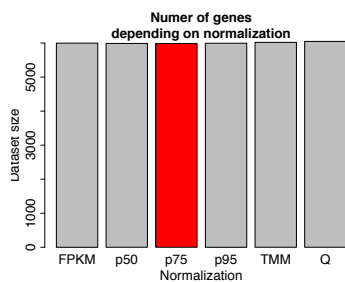

B

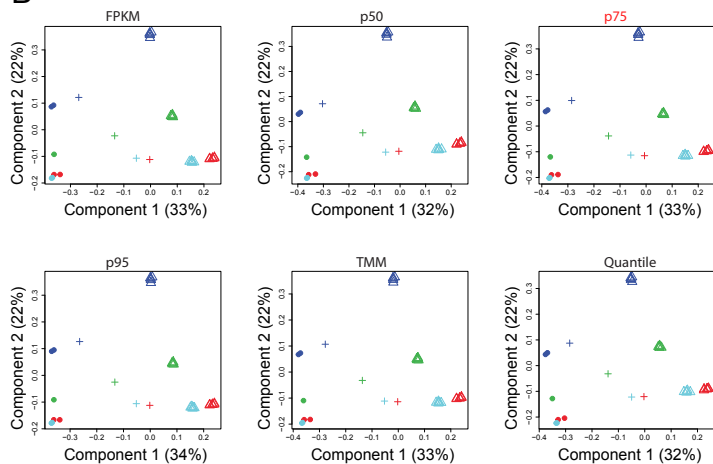

C

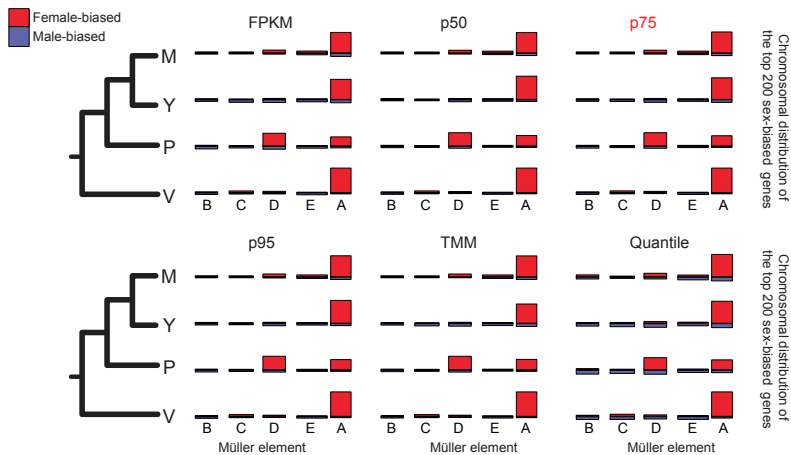

D

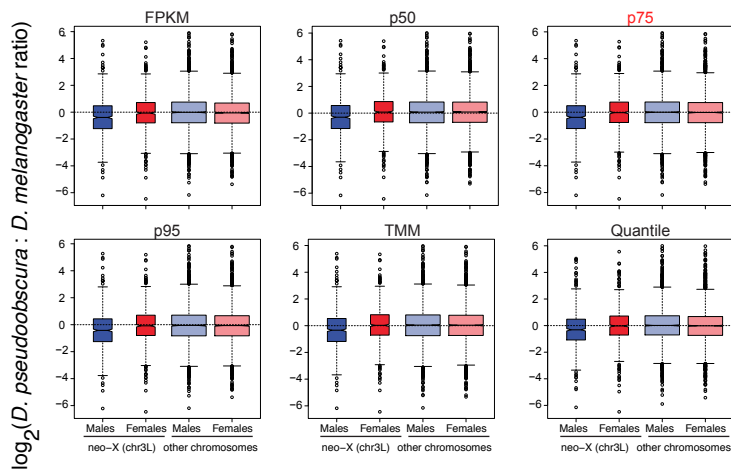

Supplement: S10 Fig — A few representative plots of the different analyses described in this manuscript are shown for all 6 normalizations tested. A. Variation in the size of the dataset, depending on normalization. p75 normalization, that was used for the main figures, is highlighted in red. B. COA analysis as described Fig 1D. C. Chromosomal distribution of sex biased genes, as described in Fig 2C. D. The increase in transcript level sex-ratio on Müller element D in D. pseudoobscura is due to a decrease in male transcript abundances specifically in D. pseudoobscura, as described in Fig 2D. (PDF) [file pgen.1005592.s010.pdf]

A

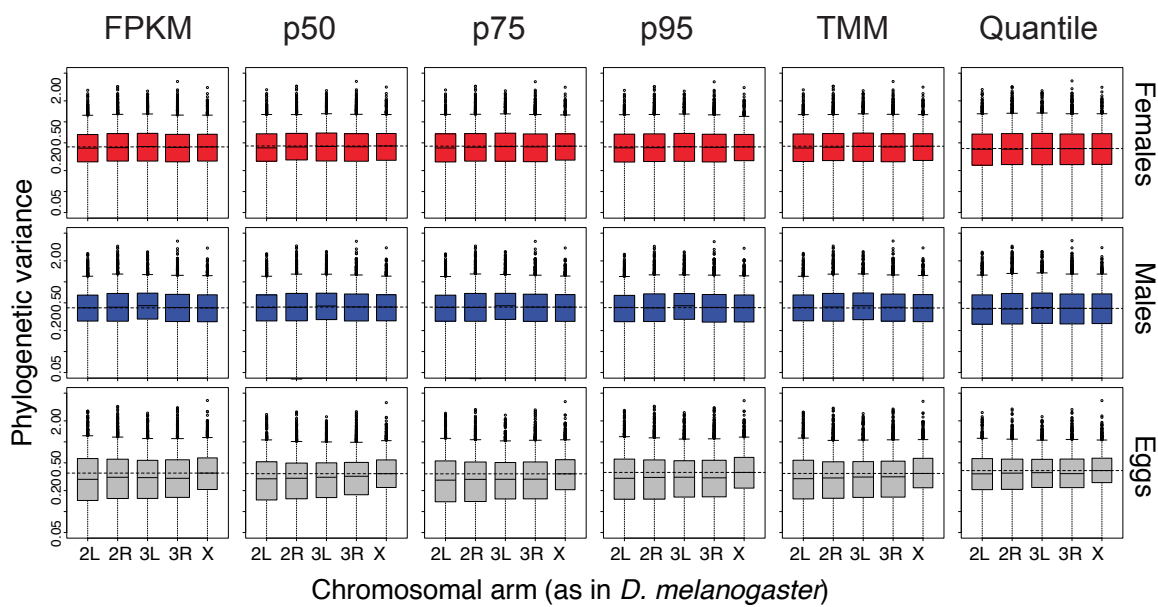

B

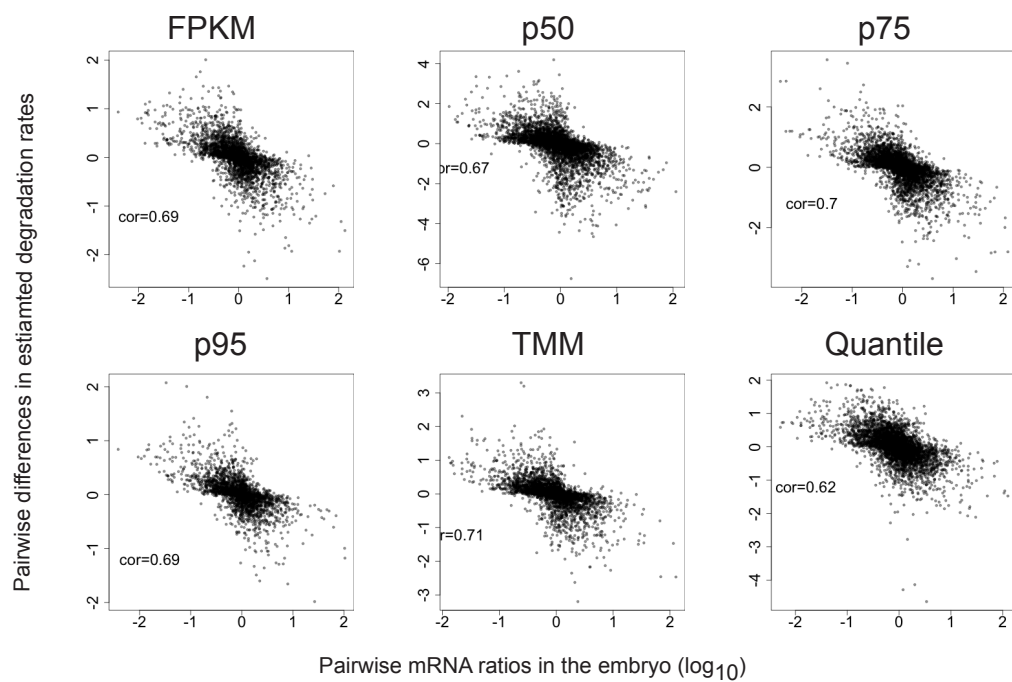

Supplement: S11 Fig — A few representative plots of the different analyses described in this manuscript are shown for all 6 normalizations tested. A. Chromosomal distribution of inter-species gene expression divergence in females, males and eggs, as in Fig 4. B. Divergence of degradation rates correlates with embryonic expression in the embryo, as described in Fig 6E. (PDF) [file pgen.1005592.s011.pdf]
