# Supplementary material for: Sex Bias and Maternal Contribution to Gene Expression Divergence in Drosophila Blastoderm Embryos
Source: PLoS Genet. 2015 Oct 20;11(10):e1005592. doi: 10.1371/journal.pgen.1005592 (PMC4618353; doi:10.1371/journal.pgen.1005592)
Supplement: S7 Fig — We compared the known classification of genes with the SVM output. Most genes maternal were predicted as maternal and almost all zygotic were predicted as zygotic. On the opposite, prediction of maternal-zygotic genes was much less successful, potentially because this group is less homogeneous. (PDF) [file pgen.1005592.s007.pdf]

Figure S7

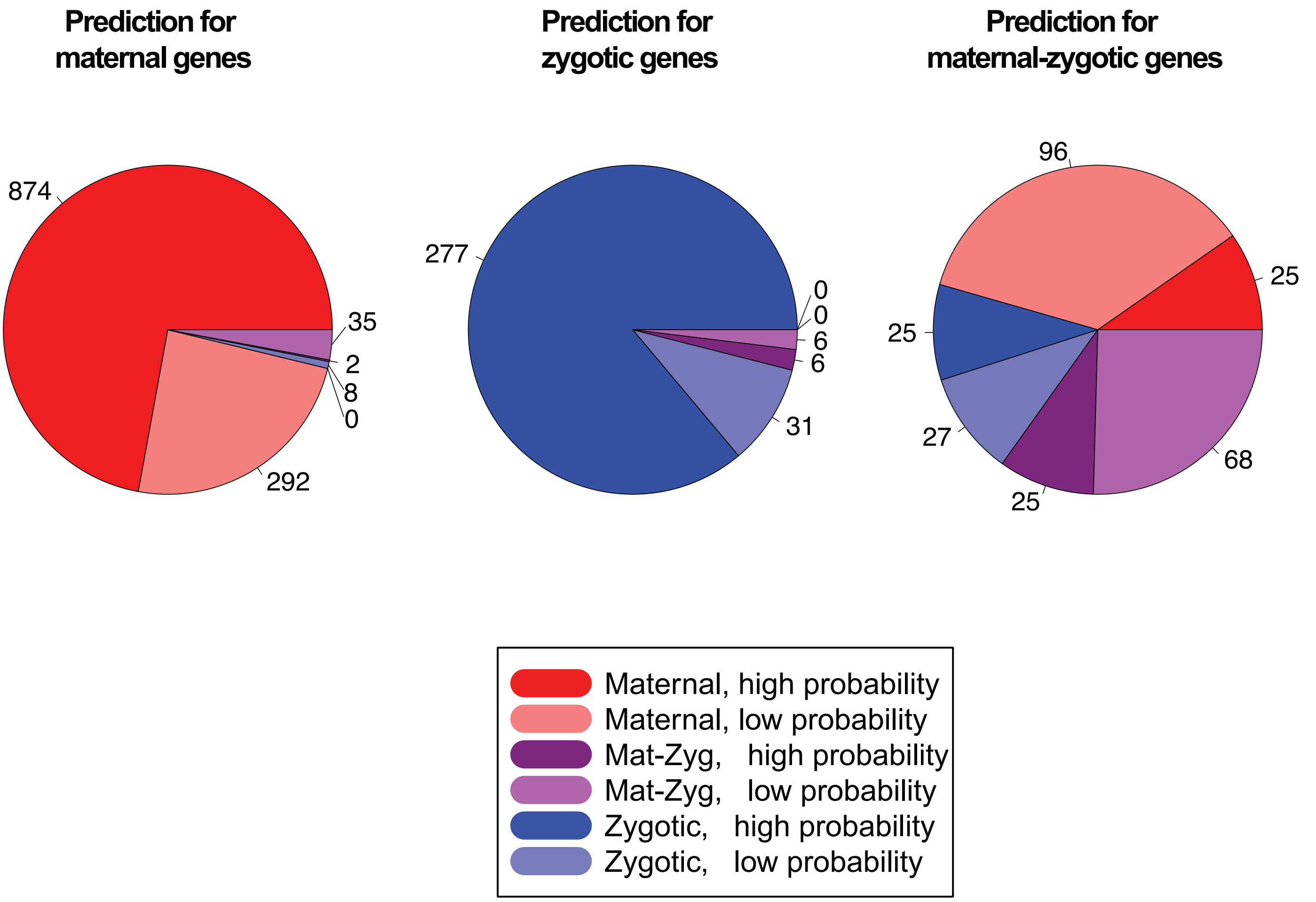

Prediction for maternal genes
874
292
35
2
8
0
Prediction for zygotic genes
277
0
0
6
6
31
Prediction for maternal-zygotic genes
96
25
25
27
25
68
Maternal, high probability
Maternal, low probability
Mat-Zyg, high probability
Mat-Zyg, low probability
Zygotic, high probability
Zygotic, low probability
